# RNA Pol II–dependent transcription efficiency fine-tunes A-to-I editing levels

**DOI:** 10.1101/gr.277686.123

**Published:** 2024-02

**Authors:** Brigitta Szabo, Therese C. Mandl, Bernhard Woldrich, Gregor Diensthuber, David Martin, Michael F. Jantsch, Konstantin Licht

**Affiliations:** Department of Cell and Developmental Biology, Center for Anatomy and Cell Biology, Medical University of Vienna, A-1090 Vienna, Austria

## Abstract

A-to-I RNA editing is a widespread epitranscriptomic phenomenon leading to the conversion of adenosines to inosines, which are primarily interpreted as guanosines by cellular machines. Consequently, A-to-I editing can alter splicing or lead to recoding of transcripts. As misregulation of editing can cause a variety of human diseases, A-to-I editing requires tight regulation of the extent of deamination, particularly in protein-coding regions. The bulk of A-to-I editing occurs cotranscriptionally. Thus, we studied A-to-I editing regulation in the context of transcription and pre-mRNA processing. We show that stimulation of transcription impacts editing levels. Activation of the transcription factor MYC leads to an up-regulation of A-to-I editing, particularly in transcripts that are suppressed upon MYC activation. Moreover, low pre-mRNA synthesis rates and low pre-mRNA expression levels support high levels of editing. We also show that editing levels greatly differ between nascent pre-mRNA and mRNA in a cellular system, as well as in mouse tissues. Editing levels can increase or decrease from pre-mRNA to mRNA and can vary across editing targets and across tissues, showing that pre-mRNA processing is an important layer of editing regulation. Several lines of evidence suggest that the differences emerge during pre-mRNA splicing. Moreover, actinomycin D treatment of primary neuronal cells and editing level analysis suggests that regulation of editing levels also depends on transcription.

Eukaryotic gene expression of protein-coding genes involves transcription, RNA processing, RNA export, translation, and degradation of transcripts (Maniatis and Reed 2002; Keene 2007; Bentley 2014; Braun and Young 2014). All of these processes are independently regulated, but interlinked to ensure tailored expression control. Recently, a growing number of evidence shows that transcripts can be modified after transcription and processing, for instance, to allow dynamic and immediate responses to stress or other external stimuli (Licht and Jantsch 2016). In analogy to epigenetic mechanisms, this phenomenon has been termed epitranscriptomics (Schwartz 2016).

One of the most prevalent epitranscriptomic processes is adenosine-to-inosine RNA editing (A-to-I editing). A-to-I editing is catalyzed by the adenosine deaminase acting on the RNA class of enzymes (ADARs), which recognize double-stranded RNA (dsRNA) as substrates. The editing reaction is a deamination of adenosine to inosine, which is then interpreted as guanosine by cellular machines (Licht et al. 2019a). The consequences of A-to-I editing are diverse and lead to recoding of transcripts, changes in splice site selection, and altered protein interactions (Rueter et al. 1999; Tajaddod et al. 2016; Licht and Jantsch 2017; Jain et al. 2019). Moreover, inosines are required to mark endogenous RNAs as “self” to the innate immune system (Mannion et al. 2014; Liddicoat et al. 2015; Eisenberg and Levanon 2018). Two catalytically active A-to-I editing enzymes, ADAR (also known as ADAR1) and ADARB1 (also known as ADAR2), have been identified in mammals. Loss of ADAR-mediated editing causes aberrant activation of the innate immune system (Mannion et al. 2014; Liddicoat et al. 2015). ADARB1 editing on the other hand targets several protein-coding transcripts in which both coding and noncoding regions can be affected (Pullirsch and Jantsch 2010; Tajaddod et al. 2016).

A major target of ADARB1 is the glutamate receptor subunit 2 (*Gria2*). The lethal phenotype of *Adarb1*^−/−^ mice can be rescued by expression of a constitutively edited version of *Gria2* (Higuchi et al. 2000). Recoding editing of the *Gria2* transcript takes place at two distinct sites called the R/G and Q/R sites and leads to recoding from arginine to glycine and glutamate to arginine, respectively. In the *Gria2* pre-mRNA, the double-stranded structure required for editing at the R/G site as well as the Q/R site is formed between an exon and the adjacent intron. Thus, editing must exclusively take place on the pre-mRNA, before removal of the intron. Editing at both sites is tightly coordinated with splicing to ensure precise regulation of the editing levels at the R/G site and the Q/R site (Schoft et al. 2007; Penn et al. 2013). Editing levels at the Q/R site need to exceed 90% in the mature RNA, as expression of the unedited Q version of *Gria2* leads to elevated Ca^2+^ influx into cells, causing cytotoxicity (Higuchi et al. 2000). Hypoediting of the Q/R site has also been associated with a series of neurological disorders such as amyotrophic lateral sclerosis (ALS), ischemia, and epilepsy (Tariq and Jantsch 2012; Jain et al. 2019). Precise regulation of editing is important not only for the *Gria2* Q/R site but for other targets as well, including the transcripts coding for the serotonin receptor (*Htr2c*), the antizyme inhibitor (*Azin1*), the insulin-like growth factor binding protein 7 (*Igfbp7*), or filamin, alpha (*Flna*) (Godfried Sie et al. 2012; Chen et al. 2013; Lyddon et al. 2013; Jain et al. 2018).

The bulk of exonic A-to-I editing occurs cotranscriptionally before pre-mRNA splicing, as recoding-editing frequently depends on an intronic editing-complementary site (Rodriguez et al. 2012; Hsiao et al. 2018). Here, we systematically study regulation and fine-tuning of editing levels across transcription, pre-mRNA splicing, and export.

## Results

### The transcription factor MYC stimulates editing levels by down-regulating transcript expression

To test if manipulation of transcription efficiency causes A-to-I editing level changes, we used a published data set from a study of transcriptional dynamics upon MYC activation using an inducible mouse fibroblast cell line (De Pretis et al. 2017). MYC activation was moderate, leading to similar expression levels as in cancer cells (De Pretis et al. 2017). The data set encompasses RNA-seq data from nascent RNA and rRNA-depleted total RNA at 11 different time points ranging from 10 min to 16 h.

After mapping the RNA-seq data to the mouse genome, we determined editing levels at known mouse editing sites that we had previously identified (Licht et al. 2019b). Five hundred eight sites were edited across all time points in nascent and total RNA (Supplemental Table S1). Before activation of MYC expression, nascent editing levels were at 6.6%. Activation of MYC expression led to 9.2% editing at time point 10 min. The editing levels for 12 sites change significantly (*P*-value = 0.05) after activation. These 12 sites mostly locate to exonic regions or UTRs. Editing levels at time point 0 min are mostly <1% and increase post activation of MYC (Supplemental Table S2). The mean editing level for nascent editing across all time points was 9.2%, whereas the mean editing level for total editing across all time points was 10.4%.

**Figure 1.**
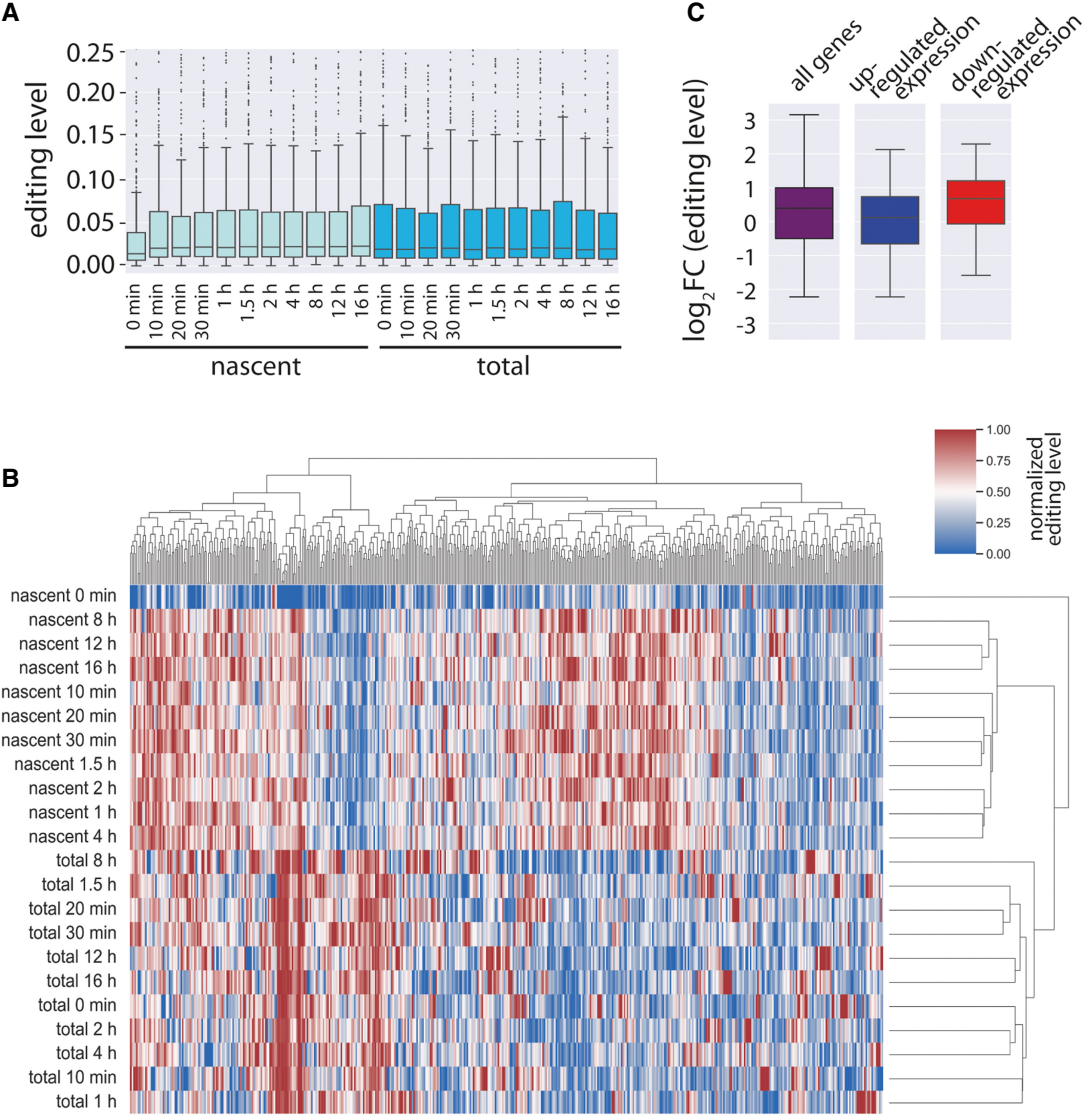
Analysis of editing in nascent and total RNA following activation of the transcription factor MYC reveals an up-regulation of editing levels upon MYC activation. Analysis of editing levels in a published data set in which nascent and total RNA were sequenced at 11 different time points following MYC activation. (*A*) Box plot showing median editing levels for all editing sites detected in the data set. (*B*) Editing levels were normalized across the entire time course and clustered using an unsupervised hierarchical clustering approach. Supplemental Table S2 shows all the editing sites sorted accordingly to the figure with individual gene names and editing site coordinates. (*C*) Log_2_ fold-change in editing levels between time point 0 min and 10 min after MYC activation for all the editing sites intersected with pre-mRNA expression data (all; purple). Those sites were split into sites associated with up-regulated (blue) or down-regulated (red) genes.

Although editing levels strongly changed between time point 0 min and time point 10 min in nascent RNA, we did not observe any apparent changes in between the other time points (Fig. 1A). However, we reasoned that distinct changes for individual sites might still be detectable. Therefore, we used an unsupervised hierarchical clustering approach including editing levels for all sites across all individual time points in nascent and total RNA (Fig. 1B; Supplemental Table S3). The tested editing sites primarily clustered into nascent and total RNA editing time points. This suggested that editing levels for individual sites differ strongly between nascent and total transcripts. In addition, nascent editing levels also clustered across individual time points. The largest difference was observed between time point 0 min (no MYC activation) and all other time points ranging from 10 min to 16 h after MYC activation. In addition, the other time points also clustered in a time-wise manner, including a rather early group (10 min, 20 min, 30 min, and 1.5 h), a middle group (1, 2, 4 h), and a late group (8, 12, 16 h). This indicates that MYC-driven changes in editing levels also occur beyond time point 10 min in a time-wise manner, specific for different editing sites.

To test if the observed up-regulation of editing levels is a direct consequence of MYC activation, we intersected our editing data set with the published MYC data set in which the investigators had determined several parameters, including pre-mRNA expression, mRNA expression, mRNA degradation, and pre-mRNA synthesis rates (Supplemental Table S4). For time points 0 min and 10 min, editing and pre-mRNA expression information was available for 777 editing sites. Of those, 243 (31%) sites were associated with up-regulated genes and 115 (15%) sites resided in down-regulated genes. This implies that ∼46% of all sites traceable in the analyzed data set reside in transcripts that are affected by MYC activation. A strong up-regulation of editing levels in nascent RNA at time point 0 min and at time point 10 min was found in transcripts down-regulated upon MYC activation (Fig. 1C). Editing levels in up-regulated genes changed only moderately. This suggests that MYC activation induces editing primarily by decreasing transcript expression, likely via affecting synthesis rates of those transcripts.

### Low synthesis rates and pre-mRNA expression levels support higher editing levels

**Figure 2.**
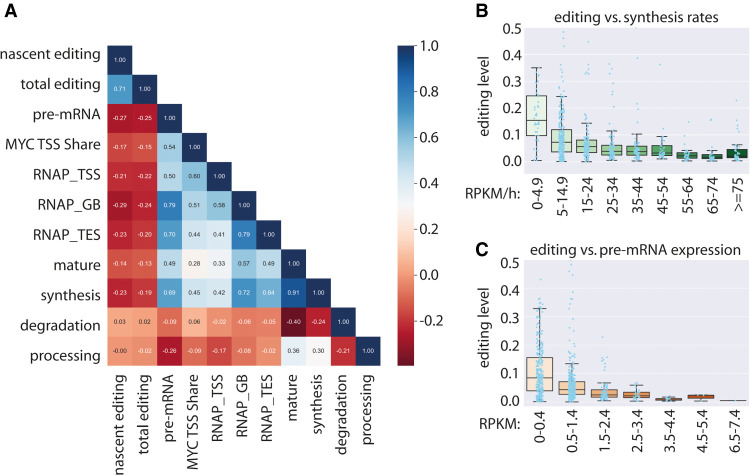
Low synthesis rates and pre-mRNA expression levels support higher editing levels. (*A*) Heatmap displaying correlation coefficients for nascent editing levels (nascent editing), total editing levels (total editing), pre-mRNA expression (pre-mRNA), MYC signal at transcription start sites (MYC TSS Share), RNA Pol II signal at transcription start sites (RNAP_TSS), gene body regions (RNAP_GB), transcription end sites (RNAP_TES), mRNA expression (mature), synthesis rates (synthesis), degradation rates (degradation), and processing rates (processing). (*B*,*C*) Nascent editing sites grouped according to synthesis rates (*B*) and pre-mRNA expression levels (*C*). Boxplots depict median editing levels and inter-quartile range. Light blue dots indicate individual editing sites. Individual editing sites above an editing level of 0.5 are not shown.

Having seen that MYC activation substantially affected editing levels via changing transcript expression, we next explored whether this observation is a more general phenomenon using all editing sites irrespective of MYC activation. Thus, we correlated gene expression and other parameters from the combined data set, including pre-mRNA expression, mRNA expression, degradation, and synthesis rates with nascent and total RNA editing levels (Fig. 2A). In particular, nascent editing levels were slightly negatively correlated with pre-mRNA expression levels (Pearson correlation coefficient −0.27) and synthesis rates (−0.23). This suggests that reduced synthesis rates and pre-mRNA expression levels lead to elevated editing. Moreover, mRNA degradation did not correlate with editing levels (Pearson correlation coefficient 0.02).

The majority of editing occurs cotranscriptionally (Rodriguez et al. 2012; Hsiao et al. 2018). Therefore, we decided to explore the correlation between editing, pre-mRNA expression, and synthesis rates, in more detail. We grouped and sorted editing sites according to the corresponding transcript synthesis rates and pre-mRNA expression levels (Fig. 2B,C; Supplemental Fig. S1). Although the bulk of editing sites in transcripts with synthesis rates between zero and 4.9 RPKM/h was edited to a maximum of ∼35%, the editing levels dropped to a maximum of 25% for synthesis rates between 5 and 14.9 RPKM/h. Higher synthesis rates of 50 or 60 RPKM/h were associated with editing levels between zero and 10% or between zero and 5%, respectively. Moreover, the majority of transcripts with low pre-mRNA expression levels between zero and 0.49 RPKM were edited to a maximum of ∼30%. The number dropped further, to a maximum of ∼15% and 7% editing, for editing sites residing in genes with pre-mRNA expression levels of 0.5–1.49 and 1.5–2.49, respectively. Importantly, synthesis rates and pre-mRNA expression levels are not analogous, which is also supported by a correlation coefficient of 0.69 between synthesis rates and pre-mRNA expression levels. This suggests that both low synthesis rates and low pre-mRNA expression levels contribute to high editing levels. Consistently, other factors that had a positive correlation with gene expression including MYC signal at promotor regions or RNA Pol II signal at transcription start sites, gene body regions, or transcription termination sites were also slightly negatively correlated with nascent editing levels (Fig. 2A). Taken together, low synthesis rates and reduced pre-mRNA expression levels supported higher editing levels, whereas high synthesis rates and enhanced pre-mRNA expression levels were linked to low editing levels. To determine whether editing would be solely driven by promoter strength, we created reporter constructs for the edited transcripts *Flna*, *Cyfip2*, *Gabra3*, and *Gria2* and cloned them downstream from a firefly reporter that was under the control of either a strong CMV promoter or a weak pGK promoter. The reporter constructs were transfected into HEK293 cells harboring or lacking a doxycycline-inducible FLAG-tagged *Adarb1* (ADAR2) transgene. qPCR indicated that expression from the CMV promoter was typically 10–20 times stronger than expression from the pGK promoter (Supplemental Fig. S2A). Determination of editing levels of the expressed pre-mRNA showed that editing did not change significantly in most cases (Supplemental Fig. S2B). However, a strong increase in pre-mRNA editing was observed for *Gabra3* when expressed from a pGK promoter rather than a CMV promoter. The same trend was observed for the *Cyfip2* reporter gene, although this increase was not significant. Still, the notion that promotor strength is the sole driver of editing levels cannot be generalized.

We also tested whether RNA degradation and editing would correlate in the MYC data set. However, neither nascent nor total RNA editing correlated with RNA degradation (Pearson correlation coefficient 0), suggesting that A-to-I editing in general does not influence the stability of transcripts. Finally, nascent and total RNA editing levels did correlate with a Pearson correlation coefficient of 0.71. Of all 508 editing sites consistently identified throughout all nascent and total time points, 215 sites changed significantly (*P*-value < 0.05, *t*-test comparing the average editing of all nascent and total time points) between nascent RNA and mRNA. Of those 215 sites, 121 had higher editing levels in nascent RNA and 94 had higher editing levels in total RNA (Supplemental Table S1).

### Amplicon sequencing of pre-mRNA and mRNA reveals strong differences in editing levels across a panel of conserved editing targets in situ

Having seen the strong differences between nascent and total RNA editing levels in the published MYC data set, we decided to compare editing of unspliced RNA and mRNA using mouse tissues. Notably, we separately tested pre-mRNA and mRNA editing levels, whereas in the MYC data set, nascent RNA editing levels and total RNA editing levels were compared. Moreover, we performed the analysis for a comprehensive panel of conserved editing sites in protein-coding regions of transcripts (Pinto et al. 2014). We focused on protein-coding targets, as a tight control of editing levels seems particularly important for those transcripts (Jain et al. 2019). To separately test editing levels for pre-mRNA and mRNA, we chose an amplicon-seq approach and designed primers specific for pre-mRNA and mRNA. Each primer contained a gene-specific sequence and an adaptor sequence, allowing a second amplification step to introduce barcoded Illumina adaptors. We isolated nascent RNA and total RNA from mouse brains at 2 wk of age, in triplicate. Following cDNA generation, the nascent RNA was used to amplify pre-mRNA, and the corresponding total RNA served as a template for the mRNA. RT-minus controls were included to control for contamination with genomic DNA. Subsequently, we isolated and pooled PCR products and submitted them to Illumina sequencing by synthesis on a HiSeq 2500 machine in a paired-end mode with 125-bp read length. Following quality control, the reads were mapped to the mouse genome, and editing levels for known sites were calculated.

**Figure 3.**
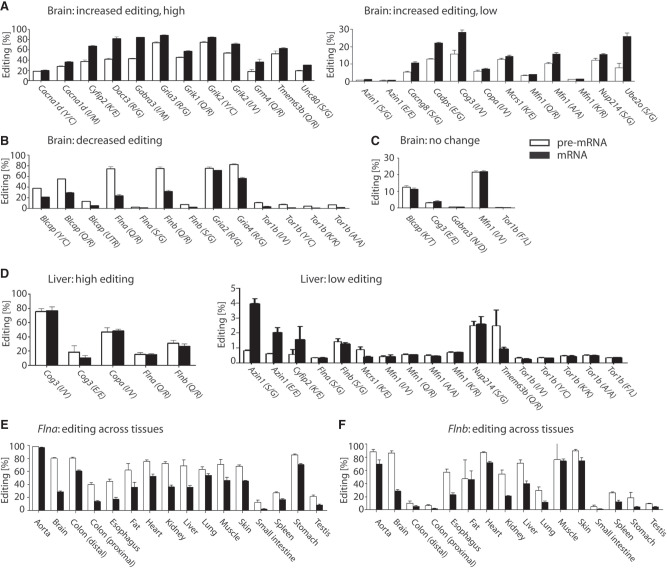
Pre-mRNA and mRNA editing levels differ across a panel of conserved protein-coding editing targets. Nascent and corresponding total RNA were isolated from mouse (2 wk of age) brain (*A*–*C*) or liver (*D*), or from a set of tissues of 8-wk-old mice (*E*,*F*). Pre-mRNA and mRNA were converted to cDNA followed by amplicon-seq. (*A*) Increased editing in mRNA versus pre-mRNA. (*Left*) Substrates with intrinsically high editing levels. (*Right*) Substrates with intrinsically low editing levels. (*B*) Decreased editing in mRNA versus pre-mRNA. (*C*) A set of editing sites with identical pre-mRNA and mRNA editing levels. (*D*) Pre-mRNA and mRNA editing in liver. (*Left*) Substrates with intrinsically high editing. (*Right*) Substrates with intrinsically low editing. (*E*,*F*) Editing levels of *Flna* or *Flnb* pre-mRNA and mRNA across different tissues. White bars indicate pre-mRNA; black bars, mRNA (n = 4 or 5).

For the majority of editing targets, we observed strong differences in editing levels for pre-mRNA and mRNA (Fig. 3A–C). Out of 42 tested editing sites, 37 sites showed significant differences in editing levels. In 10 cases, mRNA editing was at least 1.5-fold higher than pre-mRNA editing. Similarly, for 11 cases, we observed at least 1.5-fold higher editing in the pre-mRNA. Thus, analogous to the analysis in the MYC data set, editing levels can increase but also decrease from pre-mRNA to mRNA.

Editing levels are known to vary in a tissue-specific manner (Stulić and Jantsch 2013; Tan et al. 2017; Czermak et al. 2018). Therefore, we next tested if pre-mRNA/mRNA editing differences show tissue-specific patterns. Thus, we isolated nascent and total RNA from mouse liver taken at the same age and determined editing levels for 22 editing targets in the pre-mRNA and corresponding mRNA (Fig. 3D). As expected, overall editing levels differed between tissues. For instance, *Copa* mRNA editing was 7% in the brain and 48% in the liver, whereas *Flna* Q/R mRNA editing was 24% in the brain and only 15% in the liver. However, we also observed remarkable differences for editing level changes from pre-mRNA to mRNA. For instance, although in the brain pre-mRNA to mRNA editing levels of *Flna* and *Flnb* clearly dropped from 74% and 75% to 24% and 32%, respectively, in the liver, *Flna* and *Flnb* editing levels for pre-mRNA and mRNA only moderately changed from 31% to 26%. Similarly, *Cog3* I/V editing in the brain increased from 16% in pre-mRNA to 28% in mRNA and remained rather unchanged in the liver (75% and 77% for pre-mRNA and mRNA, respectively). A particularly interesting example is *Azin1*. Although *Azin1* pre-mRNA/mRNA editing levels were hardly different in the brain, the increase in editing for *Azin1* mRNA compared with pre-mRNA was about fivefold in the liver. *Azin1* is edited by ADAR-p150, which is active in the cytoplasm. Therefore, the increase is likely due to cytoplasmic editing of *Azin1* (Costa Cruz et al. 2020).

Very strong differences in pre-mRNA and mRNA editing were observed in the case of *Flna* and *Flnb* (Fig. 3B). Thus, we chose *Flna* and *Flnb* to test if differences in pre-mRNA and mRNA editing levels vary across multiple tissues as both targets are expressed and edited throughout all tissues (Stulić and Jantsch 2013; Czermak et al. 2018). To do so, we tested editing levels by amplicon-seq in pre-mRNA and mRNA of different mouse tissues isolated at 8 wk of age. We directly amplified pre-mRNA and mRNA from total RNA using specific primers. Following amplicon sequencing, we calculated editing levels (Fig. 3E,F). First, we compared the brain editing levels of *Flna* and *Flnb* from the initial analysis performed on 2-wk-old mice and the current analysis using mice of 8 wk. At 2 wk of age, the *Flna*/*Flnb* pre-mRNA editing levels were 74% and 75%, respectively, whereas mRNA editing was at 24% and 32%. In 8-wk-old mice, a moderate increase in pre-mRNA editing levels of 80% and 86% was observed for *Flna* and *Flnb* pre-mRNA, respectively. *Flna*/*Flnb* mRNA editing was 29% in each case. This moderate increase is consistent with the notion that editing levels generally increase during development (Ekdahl et al. 2012). In the mouse liver, the change is remarkable. At 2 wk of age, *Flna* pre-mRNA and mRNA editing levels were at 15%, and *Flnb* pre-mRNA and mRNA editing levels were 31% and 26%, respectively. At the age of 8 wk, *Flna* pre-mRNA and mRNA editing was 69% and 36%, respectively, whereas *Flnb* pre-mRNA and mRNA editing was 69% and 40%, respectively. For both targets, pre-mRNA editing is higher than mRNA editing across all tissues, including aorta, for which *Flna* mRNA editing levels were almost as high as pre-mRNA editing levels.

Taken together, comparing *Flna* and *Flnb* editing levels between pre-mRNA and mRNA in the brain and liver, but also across different tissues, suggests that the factors and mechanisms causing differences between pre-mRNA and mRNA editing levels show clear tissue-specific differences.

### Editing levels of spliced mRNAs moderately differ between nucleus and cytoplasm

**Figure 4.**
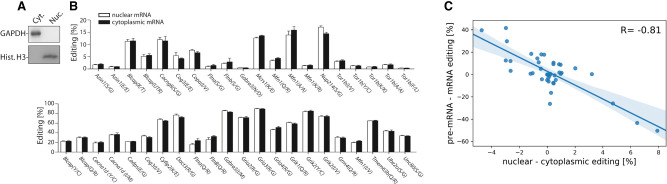
Nuclear–cytoplasmic fractionation reveals small, yet significant discrepancies in nuclear and cytoplasmic mRNA editing levels. Mouse brain (n = 5) was isolated, homogenized, and subjected to nuclear–cytoplasmic fractionation. (*A*) Nuclear (Nuc.) and cytoplasmic (Cyt.) fractions were probed with antibodies against cytoplasmic GAPDH or nuclear histone H3 (Hist. H3). (*B*) Edited transcripts were amplified from nuclear or cytoplasmic RNA, and editing levels were determined using amplicon-seq. White bars indicate nuclear mRNA; black bars, cytoplasmic mRNA. (*C*) The ratio of pre-mRNA to mRNA (pre-mRNA–mRNA) editing was plotted against the ratio of nuclear to cytoplasmic (nuclear–cytoplasmic) mRNA editing.

Conceptually, differences between pre-mRNA and mRNA editing may be explained by different splicing rates of edited or unedited transcripts, by selective nuclear export, or by different turnover of edited or unedited RNAs. However, we did not observe any correlation between mRNA stability and editing levels (Fig. 2A). To test for selective nuclear export, we prepared nuclear and cytoplasmic fractionations from mouse brains and confirmed the fractionation by Western blotting for histone H3 and GAPDH (Fig. 4A). Subsequently, we isolated RNA from both fractions. Following cDNA preparation, we amplified spliced mRNA from the nuclear and cytoplasmic fraction, respectively. Using the same pipeline as described above, we subsequently determined mRNA editing levels from both cellular compartments. Out of 42 tested editing sites, 13 sites showed significant differences in nuclear and cytoplasmic mRNA editing levels (*P*-value < 0.05). In five cases, editing levels increased. In eight cases, editing levels decreased (Fig. 4B). Next, we investigated if those differences can contribute to the differences in pre-mRNA to mRNA editing. However, differences in nuclear to cytoplasmic mRNA editing ratios even appeared to contradict the observed pre-mRNA to mRNA editing differences. For instance, although *Flna* pre-mRNA editing was higher than mRNA editing, nuclear mRNA editing was lower than cytoplasmic mRNA editing, arguing against nuclear retention of edited *Flna* mRNA. We therefore systematically tested nuclear versus cytoplasmic mRNA editing for all sites and indeed found a strong negative correlation (Pearson correlation coefficient −0.81) when we compared the observed differences between pre-mRNA to mRNA editing and the differences between nuclear and cytoplasmic mRNA editing (Fig. 4C). Seemingly, this argues for rapid export of processed RNAs and suggests that the editing process is completed in most cases before all splicing and processing events are completed (see Discussion). Notably, the differences between pre-mRNA to mRNA editing ranged from about −50% to plus 40%, whereas the differences for nuclear to cytoplasmic mRNA editing only ranged from −5% to ∼8%, showing that the impact of editing on nuclear export is rather small, yet significant.

### Editing events close to 5′ splice sites and also distant from splice sites can interfere with pre-mRNA splicing

**Figure 5.**
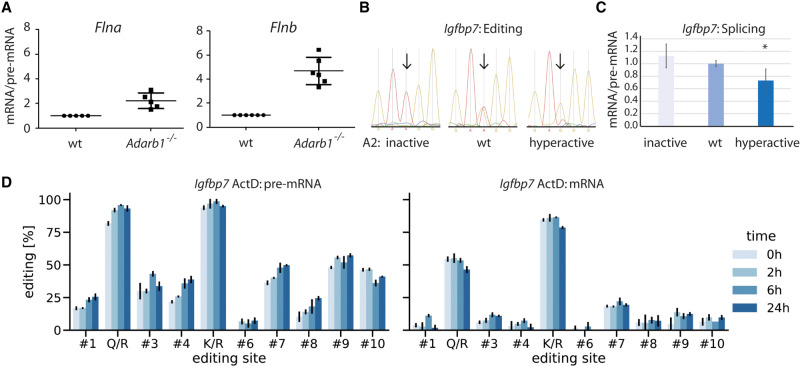
Editing sites close to 5′ splice sites and also distant from splice sites interfere with mRNA splicing. (*A*) RNA was isolated from wild-type and *Adarb1*^−/−^ mice. Subsequently, pre-mRNA splicing efficiency was determined using qPCR for the editing targets *Flna* and *Flnb*. n = 5. (*B*,*C*) An *Igfbp7* reporter construct was cotransfected into HEK293 cells either in combination with a construct expressing inactive ADARB1, a wild-type ADARB1, or a hyperactive version of ADARB1. Following isolation of RNA, editing levels (K/R site) were determined (*B*) and splicing efficiencies of the transcript in the presence of either ADARB1 was calculated (*C*). (*D*) An *Igfbp7* construct was cotransfected with wild-type ADARB1 into HEK293 cells. Subsequently, transcription was blocked with actinomycin D (ActD). Using Sanger sequencing, editing levels for pre-mRNA and mRNA were determined at time points 0 h, 2 h, 6 h, and 24 h after ActD treatment (n = 3, error bars = standard deviation).

As differences in nuclear export cannot explain the observed differences between pre-mRNA and mRNA editing levels, we next tested if differential splicing can explain the differences in editing levels. We had previously shown that ADARB1-mediated editing of *Flna* causes intron retention, presumably because editing reduces U1 snRNA base-pairing efficiency (Kapoor et al. 2020). Thus, we expanded our previous analysis and determined the splicing efficiency of *Flna* and *Flnb* in the absence of *Adarb1* using brain tissue taken from *Adarb1*^−/−^ (Fig. 5A). Although splicing efficiency for *Flna* was about twofold higher in the absence of ADARB1-mediated editing, the splicing efficiency for *Flnb* increased more than fourfold upon loss of ADARB1-mediated editing. This is remarkable as the editing site in both cases is exactly at the same position relative to the 5′ splice site and the sequence context is the same. In sum, this shows that edited *Flna* and *Flnb* are spliced less efficiently in brain and also explains why editing levels are higher for pre-mRNA compared with mRNA.

Next, we analyzed if the *Flna* or *Flnb* pre-mRNA expression levels correlate with the respective pre-mRNA or mRNA editing levels (Supplemental Fig. S3A–D). Although *Flna* pre-mRNA expression levels were positively correlated with pre-mRNA editing (r = 0.15; panel A) and mRNA editing (r = 0.43; panel B), *Flnb* pre-mRNA expression levels were negatively correlated with pre-mRNA editing (r = −0.32; panel C) and mRNA editing (r = −0.16; panel D). As the differences between pre-mRNA and mRNA levels vary across tissues for *Flna* and *Flnb*, we hypothesized that splicing efficiency correlates with pre-mRNA/mRNA editing ratios. The correlation coefficient was relatively low for *Flna* (r = 0.24) (Supplemental Fig. S4A) and *Flnb* (r = 0.21) (Supplemental Fig. S4B). However, we noticed that the splicing efficiency was particularly low for two (liver, kidney) out of 16 tissues. After removing those data points and treating them as outliers, the correlation coefficients for the remaining 14 tissues were 0.67 and 0.81 for *Flna* and *Flnb*, respectively (Supplemental Fig. S4C,D). Taken together, this suggests that splicing efficiency (pre-mRNA/mRNA ratio) contributes to determining editing level ratios across most, but not all, tissues.

Next, we analyzed splicing efficiencies in wild-type and *Adarb1*^−/−^ mice for 14 additional ADARB1 editing targets for which we had observed strong differences between pre-mRNA and mRNA editing levels (Supplemental Fig. S5). For three targets (*Cog3*, *Cyfip2*, *Grik1*), we saw significant differences (*P*-value < 0.05). For *Cog3* and *Cyfip2,* splicing efficiency dropped in the *Adarb1*^−/−^ mice, suggesting that editing promotes splicing in these substrates. This is in agreement with higher mRNA editing levels (Fig. 3). In case of *Grik1,* lack of ADARB1 led to an increase in splicing efficiency, albeit higher mRNA editing levels compared with pre-mRNA editing levels are observed.

For the remaining 11 targets, the observed differences were not significant. Notably, in the cases tested, the editing site is rather distant from the 5′ splice site. Thus, the impact of editing on splicing might be more subtle. Therefore, we turned to a system that can be manipulated more easily. We used an editable and spliceable mini-gene substrate derived from the editing substrate *Igfbp7* that we had used previously (Licht et al. 2016). In the construct, *Igfbp7* exon 1, including a part of the downstream intron, is fused to the last 60 nucleotides of intron 1 plus exon 2 from the adenovirus major late transcript (Supplemental Fig. S6). Most importantly, we had already shown that editing levels between pre-mRNA and mRNA transcribed from the reporter differ. Pre-mRNA editing levels were higher than mRNA editing levels, suggesting that the edited pre-mRNA is not spliced as efficiently as the unedited pre-mRNA. To test this, we cotransfected the *Igfbp7* construct together with a construct either expressing an editing-inactive FLAG-rADARB1, wild-type FLAG-rADARB1, or a hyperactive version of FLAG-rADARB1 into HEK293 cells (Fig. 5B,C). As expected, editing levels progressively increased for the wild-type and the hyperactive version of ADARB1. Next, using qPCR, we could show that splicing was reduced upon cotransfection of editing-active wild-type ADARB1 and was even less efficient when the hyperactive version of ADARB1 was cotransfected, showing that editing reduces splicing efficiency in the reporter construct (Fig. 5C). To confirm this and to exclude the possibility that edited mRNAs are differentially degraded, we decided to separately determine editing levels for pre-mRNA and mRNA under conditions in which transcription was blocked by actinomycin D treatment. We isolated RNA at 0 h, 2 h, 6 h, and 24 h after treatment. Subsequently, we used Sanger sequencing to determine editing levels for several sites in the pre-mRNA and mRNA of *Igfbp7*, showing that editing levels are in general higher for the pre-mRNA (Fig. 5D). Mostly, mRNA editing levels did not change over time (Fig. 5D; Supplemental Fig. S7). However, we measured a significant change for six time points in four editing sites when we compared time point 0 h to the other time points. Of those six time points, five increased compared with time point 0 h, whereas editing for one time point decreased.

In contrast, analysis of the pre-mRNA revealed significant differences for nine editing sites when comparing time point 0 h to the other time points of the respective editing site. Editing levels increased for a total of 17 time points. The increase was particularly evident at the Q/R site and the editing sites 1, 4, 6, 7, and 9.

Taken together, editing levels increase only slightly, but more strongly in pre-mRNA compared with mRNA. This supports the notion that for *Igfbp7*, edited RNA is accumulated as pre-mRNA and is spliced with reduced efficiency.

### Differences in pre-mRNA to mRNA editing levels are caused by differential splicing of edited pre-mRNAs and are linked to transcription

We next extended the analysis to several targets. Using amplicon sequencing, editing level changes were followed under conditions in which transcription was blocked. As many of the protein-coding editing targets are primarily expressed in the brain, we isolated primary neurons from mouse embryos at embryonic day (E) 12.5 and cultured them as described before (Licht et al. 2016). Following treatment with actinomycin D, we isolated RNA at time points 0 h, 6 h, and 24 h after treatment and determined transcript abundance. As expected, RNA abundance dropped after 6 h and 24 h, showing that transcription was successfully blocked (Supplemental Fig. S8). Because of the limited amount of primary neuronal cells, we could not enrich for nascent RNA but amplified pre-mRNAs without prior enrichment. In total, we amplified pre-mRNAs and mRNAs from 11 transcripts, encompassing 16 editing sites (Supplemental Fig. S9). Notably, compared with the analysis performed using mouse tissues, editing levels for individual biological replicates were rather heterogenous, as seen before (Licht et al. 2016). Nevertheless, at time point 0 h, the change in editing between pre-mRNA and mRNA showed the same trend as for mouse brain for 15 out of 16 editing sites, with the exception of *Cacna1d* (Supplemental Fig. S9). This suggests that primary neuronal cells mimic the situation in brain tissue very well. Overall, editing levels in pre-mRNA and mRNA of the analyzed transcripts increased over time.

**Figure 6.**
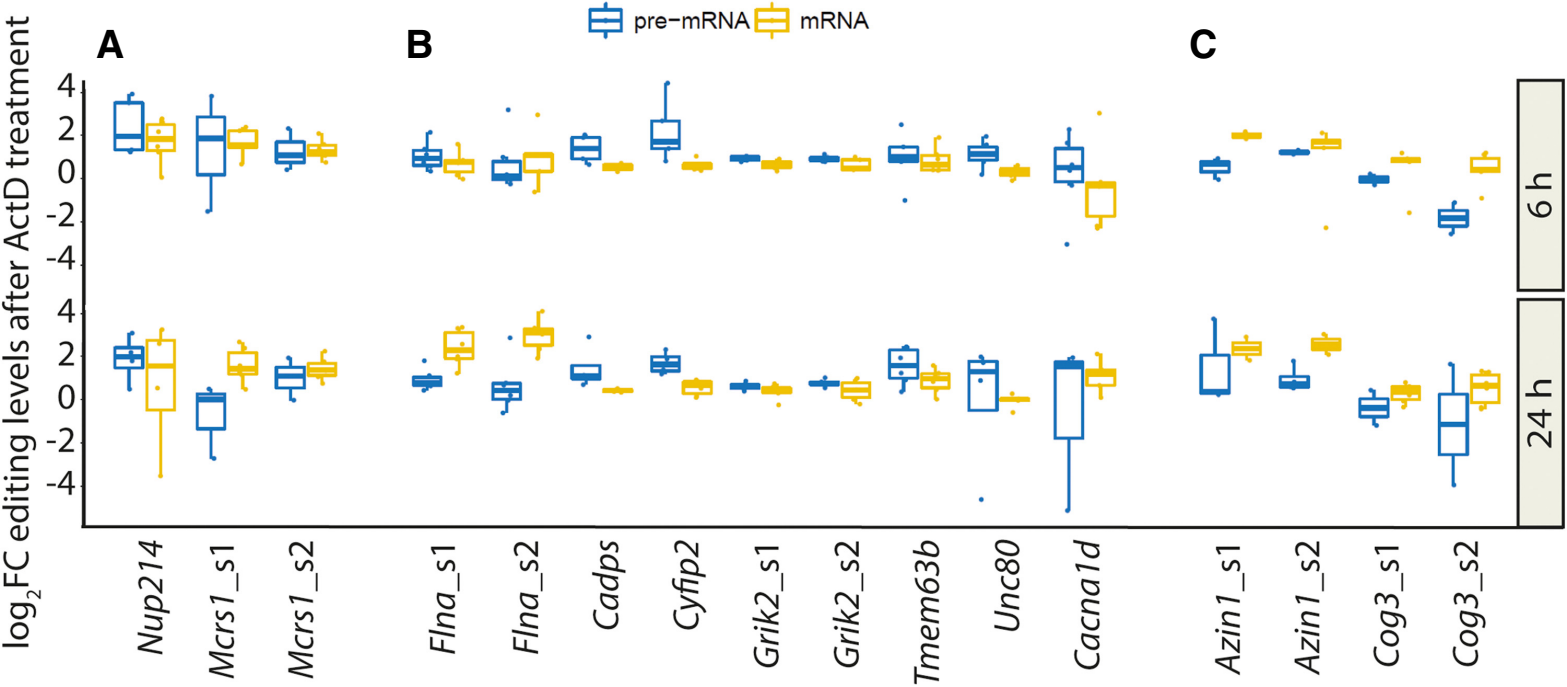
Differences in pre-mRNA and mRNA editing levels are reverted under conditions in which transcription is blocked. (*A*–*C*) RNA was isolated from primary neuronal cultures established from wild-type mouse embryos at E12.5 that were treated with actinomycin D in order to stall transcription. Subsequently, pre-mRNA and mRNA editing levels were determined using amplicon-sequencing. The log_2_FC in editing levels for pre-mRNA (blue) or mRNA (yellow) relative to the control without treatment at 0 h is plotted for 6 h (*upper* panel) and 24 h (*lower* panel), respectively (n = 6).

However, we were primarily interested to see how the differences in pre-mRNA editing and mRNA editing changed over time. Therefore, we calculated the fold-change relative to time point 0 h for all transcripts (Fig. 6). Although the ratio of pre-mRNA to mRNA editing did not change for the transcripts *Nup214* and *Mcrs1* (especially site 2) (Fig. 6A), the majority of transcripts, including *Cacna1d*, *Cadps*, *Cyfip2*, *Grik2*, *Unc80*, and *Tmem63b*, displayed a trend toward stronger editing in pre-mRNA than in mRNA (Fig. 6B,C). Three transcripts—*Cog3*, *Flna*, and *Azin1* (Fig. 6B,C)—showed an opposing trend, with a stronger increase in mRNA editing. First, this suggests that edited transcripts are differentially spliced. Second, this means that under conditions in which transcription is blocked, the initially observed differences in pre-mRNA and mRNA editing are reverted. Under steady-state conditions, editing levels were higher for *Cadps*, *Cyfip2*, *Grik2*, *Unc80*, and *Tmem63b* mRNA than pre-mRNA (Fig. 3). Upon blocking transcription, the effect reverted, and pre-mRNA editing levels increased over mRNA editing (Fig. 6B). In the case of *Flna*, pre-mRNA editing is higher under steady-state conditions, and mRNA editing is lower. Again, blocking transcription reverted the effect (Fig. 6B). *Azin1* editing is mediated by ADAR-p150, which occurs in the cytoplasm. Notably, ADAR-p150 expression does not change upon inhibition of transcription (Supplemental Fig. S10). The underlying reason for the opposing trend seen for *Azin1* is speculative (see Discussion). The difference in editing levels for *Cog3* between pre-mRNA and mRNA is unclear as it displays different trends depending on the tissue (brain vs. liver). Importantly, as the editing-competent stem for most of the aforementioned targets is formed between intron and exon, editing cannot simply increase owing to editing of the spliced mRNA. Taken together, this suggests that the differences in pre-mRNA and mRNA editing levels are primarily caused by differential splicing of the edited RNA but are indirectly regulated by transcription.

## Discussion

The bulk of A-to-I editing occurs cotranscriptionally (Rodriguez et al. 2012; Hsiao et al. 2018). Therefore, A-to-I editing can lead to alternative splicing. For instance, the *Adarb1* transcript itself is edited, which leads to alternative splicing (Rueter et al. 1999). Another example is the glutamate receptor subunit *Gria2*. Here, editing occurs at two individual sites, which leads to alternative splicing of nearby exons (Schoft et al. 2007; Penn et al. 2013). Moreover, other studies have addressed the impact of editing on splicing more systematically on a transcriptome-wide level (St Laurent et al. 2013; Solomon et al. 2013; Hsiao et al. 2018; Kapoor et al. 2020).

Here, we discern editing regulation during transcription and pre-mRNA splicing. Several RNA-binding proteins, including alternative splicing factors, are regulators of A-to-I editing (Garncarz et al. 2013; Tariq et al. 2013; Tan et al. 2017; Quinones-Valdez et al. 2019). However, the impact of transcription regulation on A-to-I editing levels is not known. We showed that activation of the transcription factor MYC causes strong alterations in editing levels. In general, transcripts that were suppressed by MYC showed an increase in editing. This is consistent with our finding that editing levels were particularly high in transcripts with low pre-mRNA synthesis rates.

Low pre-mRNA synthesis rates and, consequently, low pre-mRNA expression levels are correlated with high editing levels in the MYC data set. Most likely, low synthesis rates allow more time for ADAR enzymes to catalyze the A-to-I editing reaction. A reporter gene experiment in which editing sites were expressed from a highly active viral promotor or a moderate promoter could only partly confirm that low synthesis rates correlate with increased editing. In the reporter gene experiment, we only focused on protein-coding transcripts that are linked to splicing. However, in the MYC data set, we looked at all editing sites including UTR-editing and intronic editing, which could explain the differences.

We also observed clear differences between pre-mRNA and mRNA editing levels in the MYC data set. Using reporter constructs, we previously observed differences between pre-mRNA and mRNA editing levels on a small set of editing targets, suggesting that editing levels are regulated during transcript maturation (Licht et al. 2016). We confirmed and precisely quantified this finding using mouse tissues and an amplicon-seq approach focusing on conserved protein-coding editing targets. Editing levels either increased or decreased from pre-mRNA to mRNA in a substrate-specific manner. In line with our finding, differences in editing levels for polyadenylated and total RNA have been observed previously in human cells (Hsiao et al. 2018). A potential drawback of the amplicon-seq approach is that we only amplified the predominant unspliced variant and cannot capture potential intermediates or alternative splicing variants. Moreover, the predominant unspliced variant may not correspond to the nascent RNA.

In the present study, we combined data from a study in mouse fibroblast cells in which the investigators compared nascent and total RNA. Our experimental data come from mouse tissues or reporter constructs. For technical reasons, we mostly compared pre-mRNA and mRNA in this case. Although nascent RNA and pre-mRNA as well as total RNA and mRNA share some properties, they are not identical. This also limits a direct comparability between data analyzed here.

It remains elusive if editing can control stability of (cytoplasmic) mRNA. Data generated using model substrates suggested that the RISC subunit Tudor-SN causes inosine-specific degradation of hyperedited substrates (Scadden 2005). This would imply that higher editing levels trigger Tudor-SN-mediated RNA degradation. However, we did not observe any general correlation between RNA degradation and editing levels, suggesting that RNA degradation of inosine-containing RNAs plays a negligible role and may be specific to a certain set of substrates, like certain hyperedited transcripts.

We provide two direct lines of evidence that editing can interfere with splicing. First, *Flna* and *Flnb* pre-mRNAs are more efficiently spliced in mice lacking the editing-active ADARB1 protein. Second, a model substrate of *Igfbp7* is less efficiently spliced when edited to higher extents. Importantly, the editing sites of *Flna* and *Flnb* are located at position −2 relative to the 5′ splice site, suggesting that editing might directly interfere with spliceosome assembly. Previously, it was shown that editing in the glutamate receptor subunit *Gria2* close to the 5′ splice site leads to changes in splicing (Schoft et al. 2007). In case of the *Igfbp7* construct, the editing sites are distant from splice sites. Thus, editing at this position likely indirectly interferes with splicing, for instance, by preventing binding of accessory RNA processing factors or by causing structural rearrangements in the pre-mRNA. It was suggested previously that editing may alter RNA structures and cause changes in pre-mRNA splicing (Rieder and Reenan 2012). For many targets, we observed an increase of editing levels from pre-mRNA to mRNA, including targets like *Cyfip2*, *Gabra3*, and *Azin1*. *Azin1* is edited by ADAR-p150. Thus, cytoplasmic editing by ADAR-p150 might explain the increase of editing in the cytoplasm (Costa Cruz et al. 2020). *Gabra3* forms an editing-competent stem within the edited exon itself (Ohlson et al. 2007). Therefore, ADAR or ADARB1 can still edit the transcript following splicing. This may explain higher mRNA editing levels. Notably, editing of *Gabra3* can be enhanced by an intronic stem in the downstream exon (Daniel et al. 2012). However, we did not include this downstream intronic stem in our reporter constructs, which may explain the relatively low *Gabra3* editing levels seen in this context (Supplemental Fig. S2).

Upon blocking transcription with actinomycin D, we saw that the differences in editing levels between pre-mRNA and mRNA reverted. This observation might be explained by differences in enzyme versus substrate ratio: Although ADAR protein levels presumably will not be immediately affected by blocking transcription, the level of transcripts will drop. This might also explain why cytoplasmic mRNA editing of *Azin1* increases. *Azin1* is edited by cytoplasmic ADAR-p150. As actinomycin D treatment blocks transcription, no new unedited transcript is produced. Thus, the proportion of edited *Azin1* should increase over time.

Moreover, blocking transcription might also reduce splicing efficiency as both processes are intrinsically coupled (Khodor et al. 2011; Bentley 2014). Thereby, pre-mRNA might remain available for editing, whereas the spliced mRNA will be less affected. Additionally, coupling of RNA editing and splicing via the C-terminal domain of the RNA polymerase II might play a role (Laurencikiene et al. 2006).

We observed no systematic difference in editing levels between nuclear spliced mRNAs and cytoplasmic mRNAs. In contrast, editing levels either increased or decreased. This suggests that the edited RNAs tested here are neither retained nor preferably exported out of the nucleus. The subtle differences in editing levels for nuclear spliced RNA and cytoplasmic mRNA observed for some transcripts might be linked to transcription, as splicing and transcription are coupled and spliced RNA leads to recruitment of the exon–junction complex, which itself promotes nuclear export (Le Hir et al. 2001; Erkmann and Kutay 2004).

We saw strong negative correlation between the differences in pre-mRNA to mRNA editing levels and the differences in nuclear spliced RNAs to cytoplasmic mRNAs. An explanation might be that we enriched for transcripts that are stochastically spliced more rapidly than the bulk of transcripts, which would counterbalance the pre-mRNA to mRNA editing levels and therefore explain the anticorrelation.

## Methods

### Isolation of nascent and total RNA

Nascent and total RNA from 2-wk-old mouse brain or liver were isolated as described previously (Menet et al. 2012). The respective organ was homogenized in 3.5 mL of 1× PBS and 3.5 mL of homogenization buffer (2.2 M sucrose, 10 mM Hepes at pH 7.6, 15 mM KCl, 2 mM EDTA, 1× protease inhibitor cocktail [Roche], 0.15 mM spermine, 0.5 M spermidine, 0.5 mM DTT) with a 7-mL Dounce homogenizer (six strokes loose pestle, four strokes tight pestle). Three hundred microliters lysate was used for total RNA isolation, and the rest was then mixed with 21.5 mL of homogenization solution and layered on the top of a 10 mL ice-cold cushion solution (2.05 M sucrose, 10 mM Hepes at pH 7.6, 10% glycerol, 15 mM KCl, 2 mM EDTA, 1× protease inhibitor cocktail [Roche], 0.15 mM spermine, 0.5 M spermidine, 0.5 mM DTT) and centrifuged for 45 min at 4°C at 24,000 rpm (100,000*g*). Nuclei were resuspended in 1 mL of 20 mM Hepes (pH 7.6), 150 mM NaCl, 2 mM EDTA, 1× protease inhibitor cocktail (Roche), 1 mM DTT, 0.5 U/mL of murine RNase inhibitor (New England Biolabs); homogenized using a 1-mL Dounce homogenizer (three times with loose pestle, two times with tight pestle); and transferred to a Falcon tube. One volume of 2× NUN buffer (50 mM Hepes at pH 7.6, 2 M urea, 2% NP-40, 600 mM NaCl, 2 mM DTT, 1× protease inhibitor cocktail [Roche], 0.5 U/mL of murine RNase inhibitor [New England Biolabs]) was subsequently added drop-by-drop while gently vortexing (level 2). The samples were left on ice for 20 min and subsequently centrifuged at 15,000 rpm for 20 min at 4°C. After removal of the supernatant, 1 mL of TriFast (Peqlab) was added to the pellet. Following incubation for 15 min at 65°C, the DNA pellet was resuspended by gentle pipetting. Subsequently, nascent and total RNA were extracted according to the manufacturer's guidelines. Ten micrograms of isolated RNA was incubated with 10 units of DNase I (New England Biolabs) for 45 min at 37°C (in case of nascent RNA, 3 units of DNase I per µg RNA were used). Subsequently, RNA was phenol-chloroform-extracted. One microgram of total RNA was reverse-transcribed using random hexamer priming and M-MuLV reverse transcriptase (New England Biolabs) following the manufacturer's instructions. A control without reverse transcriptase was included for every sample.

### Isolation of total RNA from subcellular fractions

Subcellular fractionation was performed on 2-wk-old mouse brain as published previously with several modifications (Baghirova et al. 2015). The dissected brain was homogenized in ice-cold lysis buffer A (50 mM Hepes at pH 7.4, 150 mM NaCl, 100 µg/mL digitonin [Sigma-Aldrich D141], 1 M hexylene glycol [Sigma-Aldrich 112100], 1× protease inhibitor cocktail [Roche], 10 mM ribonucleoside vanadyl complex [New England Biolabs]) using a 7-mL Dounce homogenizer (six strokes loose pestle, four strokes tight pestle). One volume of lysis buffer A was added. After incubation for 10 min on an end-over-end rotator at 4°C, the homogenate was centrifuged at 4000*g* for 10 min at 4°C, and the supernatant containing the cytosolic fraction was set aside. Using a 1-mL pipette and tip, the pellet was resuspended by gently pipetting up and down in ice-cold lysis buffer B (50 mM Hepes at pH 7.4, 150 mM NaCl, 1% NP-40, 1 M hexylene glycol [Sigma-Aldrich 112100], 1× protease inhibitor cocktail [Roche], 10 mM ribonucleoside vanadyl complex [New England Biolabs]) and incubated for 30 min on an end-over-end rotator at 4°C. Lysate was centrifuged at 5000g for 20 min at 4°C, and supernatant was discarded. Nuclei pellet was resuspended in ice-cold lysis buffer C (50 mM Hepes at pH 7.4, 500 mM NaCl, 0.5% sodium deoxycholate, 0.1% sodium dodecyl sulphate, 1 M hexylene glycol [Sigma-Aldrich 112100], 1× protease inhibitor cocktail [Roche], 10 mM ribonucleoside vanadyl complex [New England Biolabs]) and sonicated. The lysate was centrifuged at 15,000 rpm for 10 min at 4°C. The supernatant serves as the nuclear fraction. RNA was isolated and reverse-transcribed as described above.

### Amplicon sequencing, library preparation, analysis, and splicing efficiency

The library preparation involved two subsequent PCR steps. Initially, the targeted genes were amplified with specific primers also containing partial Illumina adapters. In the second PCR, barcoded Illumina adapters were attached. The first and second PCR reactions were performed using 12.5 µL OneTaq 2× master mix with standard buffer (New England Biolabs) and 0.2 µM forward and reverse primer. In the first PCR, 1 µL cDNA was amplified for 25 cycles. For the second PCR, 5 µL of first PCR reaction was used and amplified for 15 cycles. Subsequently, PCR reactions were subjected to gel electrophoresis on 2% agarose gels. PCR products were excised from the gel and purified with Wizard SV gel and PCR clean-up system (Promega). The amplicons were then pooled equimolarly and purified using AMPure XP beads (Beckman Coulter). Sequenced amplicons were mapped to mm10 using HISAT2 Galaxy version 2.1.0 (Kim et al. 2015; Jalili et al. 2020). Mapping was manually inspected using the Integrative Genomics Viewer (IGV) (Thorvaldsdottir et al. 2013). Editing levels at known sites were calculated using Pysam (https://github.com/pysam-developers/pysam). The percentage of editing is calculated as the number of G divided by the sum of A plus G or as the number of C divided by the sum of T plus C. To test for splicing efficiencies, qPCR was performed using Luna universal qPCR master mix (New England Biolabs M3003) according to instructions. ΔΔCT values were calculated by comparing to *Gapdh*.

### Transfection, actinomycin D treatment, RNA isolation, RT-PCR, and Sanger sequencing

Twenty-four hours before transfection, 2 × 10^5^ HEK293 cells were seeded on six-well plates and cotransfected with 0.5 µg *Igfbp7* minigene plasmid (Licht et al. 2016)⁠ and 3.5 µg FLAG-tagged rat *Adarb1* plasmid using jetPEI (Polyplus). Subsequently, cells were treated with 5 µg/mL actinomycin D for 0, 2, 6, or 24 h. Forty-eight hours after transfection total RNA was isolated using TriFast (Peqlab) according to the manufacturer's guidelines. Seven micrograms of isolated RNA was incubated with 70 units BamHI-HF restriction enzyme (New England Biolabs) for 45 min at 37°C and, afterward, with 20 units of DNase I (New England Biolabs) for 45 min at 37°C. Subsequently, RNA was phenol-chloroform-extracted. 1 µg of total RNA was reverse-transcribed as described above.

To amplify pre- and mature mRNA Q5 polymerase with GC Enhancer (New England Biolabs) was used as follows: 98°C for 30 sec, then 35 cycles of 98°C for 10 sec, 58°C for 20 sec, 72°C for 25 sec, followed by 72°C for 2 min. In case of mRNA semi-nested PCR amplification was used. PCR products were size-selected as described above.

### Expression and editing correlation of filamin, alpha and filamin, beta pre versus mature mRNA in different tissues

After sacrifice of 8-wk old wild-type mice, the respective organ was homogenized in TriFast (Peqlab) using a bead tissue homogenizer. Beads were removed by centrifugation at 15,000 rpm for 10 min at 4°C. Total RNA was isolated from the supernatant according to the manufacturer's guidelines. 10 µg of isolated RNA was incubated with 10 units of DNase I (New England Biolabs) for 45 min at 37°C. Subsequently, RNA was phenol-chloroform-extracted, and 1 µg of total RNA was reverse-transcribed as described. To check expression levels, qPCR was performed using Luna universal qPCR master mix (New England Biolabs M3003) according to the instructions. ΔΔCT values were calculated by comparing to the geometric mean of two reference gene (*Snrpd1* and *Vcp*).

### Primary neuronal cell culturing, actinomycin D treatment, and analysis

Primary neuronal cell cultures were established and maintained as described previously (Licht et al. 2016). To stall transcription, 10 µg/mL of actinomycin D was applied to the cells for 0 h, 6 h, and 24 h, respectively. RNA was isolated and reverse-transcribed as described. qPCR was performed to assay the effect of actinomycin D treatment on RNA quantities for several transcripts. Using amplicon sequencing, pre-mRNAs and mRNAs were amplified. To analyze the data, reads were mapped to the mouse genome (mm10) using STAR (Dobin et al. 2013). Mappings were manually checked using IGV. Editing levels were quantified using Pysam. Editing levels changes were calculated and plotted using custom R (R Core Team 2021)⁠ scripts.

### MYC data analysis

Raw sequencing reads were downloaded from NCBI Gene Expression Omnibus (GEO; https://www.ncbi.nlm.nih.gov/geo/) accession number GSE98420. Following conversion to FASTQ, the reads were mapped to the mouse genome mm10. The data set contains data for 22 conditions (11 time points, nascent and total). Therefore, instead of requiring each known editing site to be covered in all 22 conditions with a minimum of number of reads, a dynamic approach without a fixed coverage was used. First, editing levels at known sites were quantified. Subsequently, all editing sites where the editing level was zero were excluded from the analysis and marked as “NA” as we reasoned that the particular site was not sufficiently covered. For the analyses in Figure 1, only editing sites edited under all 22 conditions were kept. The information from the intersected data set was only available for 52 of those editing sites. Thus, for the analyses in Figure 2, all editing sites were kept where editing was higher than zero in at least six out of all 22 conditions (nascent plus total time points). Sites not edited in a particular condition were dynamically excluded. The clustermap in Figure 1 was generated using the Python seaborn library with the settings vmin = 0, vmax = 1, metric = “minkowski, method = “complete,” standard_scale = 0.

## Data access

All raw and processed sequencing data generated in this study have been submitted to the European Nucleotide Archive (ENA; https://www.ebi.ac.uk/ena/browser/home) under accession number PRJEB58992. Jupyter Notebooks and R Scripts that were used to analyze data are available as Supplemental Code.

## Supplementary Material

Supplement 1

Supplement 2

Supplement 3

Supplement 4

Supplement 5
