# Supplementary material for: RNA Pol II–dependent transcription efficiency fine-tunes A-to-I editing levels
Source: Genome Res. 2024 Feb;34(2):231–42. doi: 10.1101/gr.277686.123 (PMC10984384; doi:10.1101/gr.277686.123)
Supplement: Supplement 1 [file Supplemental_Material.pdf]

Supplemental Material

RNA Pol-II dependent transcription efficiency fine-tunes A-to-I editing levels

Brigitta Szabo, Therese C. Mandl, Bernhard Woldrich, Gregor Diensthuber, David Martin, Michael F. Jantsch, and Konstantin Licht

Supplemental Table S2: Editing sites were editing levels changed significantly in nascent RNA when comparing time points 0 and 10 minutes post MYC activation. Genome coordinates (chr, pos) refer to mouse mm10. ENS_id = Ensembl gene ID.

chr	pos	str	annotation	gene_name	ENS_id	0_min	10_min	P-value
chr11	66878140	+	UTR	Sco1	ENSMUSG00000069844	0.18%	1.44%	0.0240
chr16	10842105	+	UTR	Rmi2	ENSMUSG00000037991	0.35%	1.49%	0.0302
chr1	174022479	+	Exon	Copa	ENSMUSG00000026553	4.17%	19.80%	0.0478
chr2	143401286	+	Exon	Pcsk2	ENSMUSG00000027419	1.50%	8.21%	0.0178
chr3	88156594	+	Exon	Smg5	ENSMUSG00000001415	0.04%	0.57%	0.0049
chr5	23666943	+	intergenic	Klhl7	ENSMUSG00000028986	0.10%	1.99%	0.0392
chr9	20823973	+	Intron	Icam1	ENSMUSG00000037405	0.71%	2.10%	0.0401
chr10	126504938	-	UTR	AC134329.1	ENSMUSG00000112639	0.08%	0.89%	0.0062
chr10	33709058	-	intergenic	AC153962.3	ENSMUSG00000112280	27.62%	71.67%	0.0432
chr17	24299404	-	Exon	Amdhd2	ENSMUSG00000036820	0.23%	0.58%	0.0413
chr3	116447000	-	UTR	Agl	ENSMUSG00000033400	0.23%	1.09%	0.0067
chr7	147996955	-	UTR	Zfp941	ENSMUSG00000060314	1.22%	5.44%	0.0334

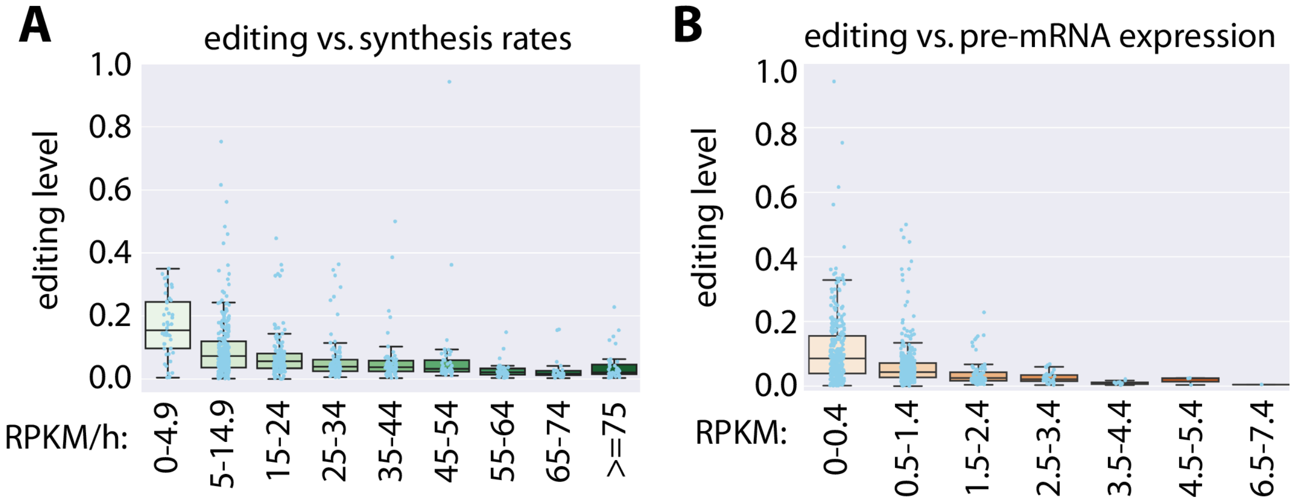

Supplemental Figure S1: Uncropped version of panels B and C from main figure 1. A, B) Nascent editing sites grouped according to synthesis rates (A) and pre-mRNA expression levels (B). Boxplots depict median editing levels and inter quartile range. Light blue dots indicate individual editing sites.

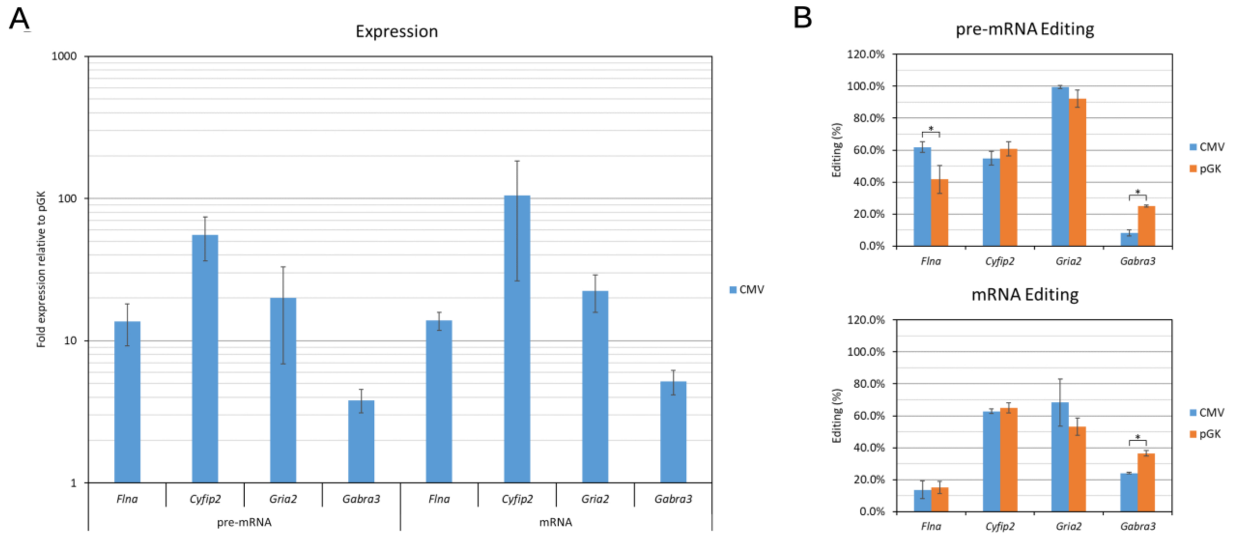

Supplemental Figure S2: pre-mRNA editing levels for editing sites under the control of a CMV or pGK promoter. Edited transcripts were cloned and put under the control of a CMV or pGK promoter. Subsequently, the reporter constructs were transfected into HEK293 cells in the presence of ADAR2. A) Expression levels of transcripts under the control of a CMV promoter relative to the corresponding transcript expressed from a plasmid with pGK promoter. Due to the logarithmic scale, pGK is not shown. B) Editing levels for pre-mRNA and mRNA. The asterisk indicates significance. n=3.

A

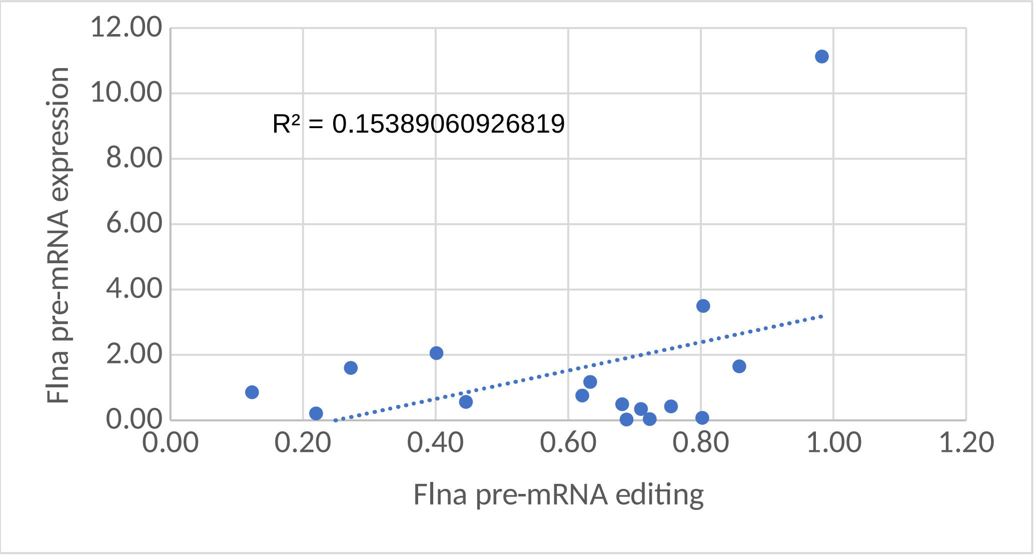

B

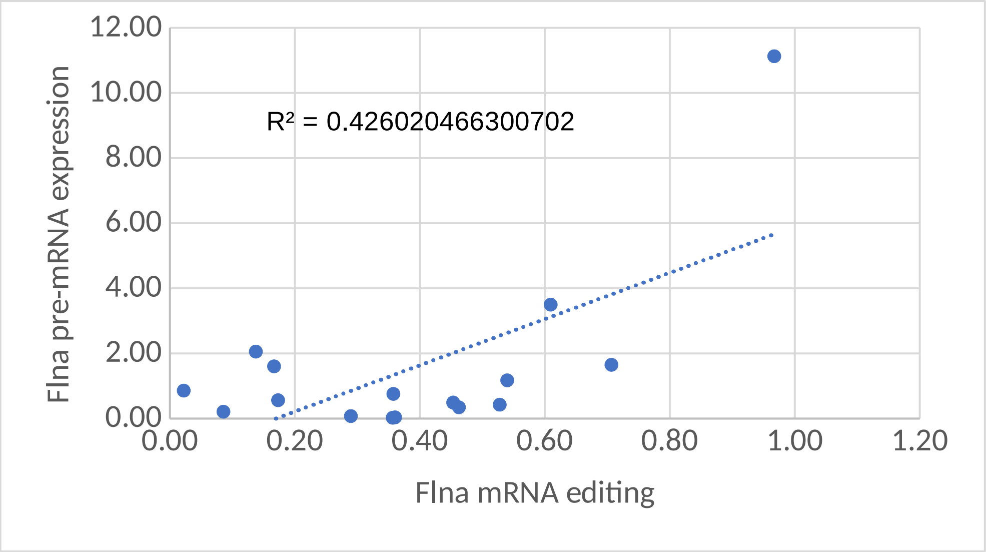

12.00
10.00
8.00
6.00
4.00
2.00
0.00
0.00
0.20
0.40
0.60
0.80
1.00
1.20
Flna pre-mRNA expression
Flna mRNA editing
R² = 0.426020466300702

C

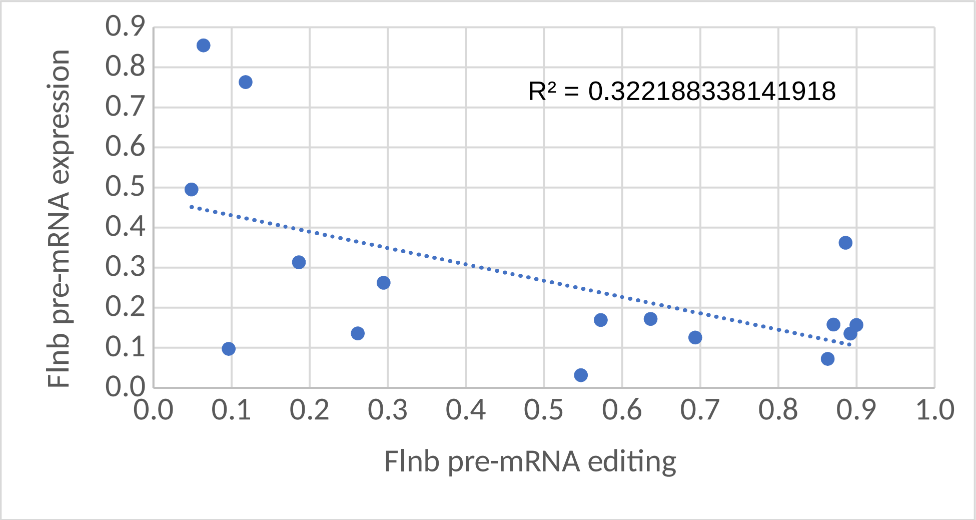

0.9
0.8
0.7
0.6
0.5
0.4
0.3
0.2
0.1
0.0
0.0
0.1
0.2
0.3
0.4
0.5
0.6
0.7
0.8
0.9
1.0
Flnb pre-mRNA expression
Flnb pre-mRNA editing
R² = 0.322188338141918

D

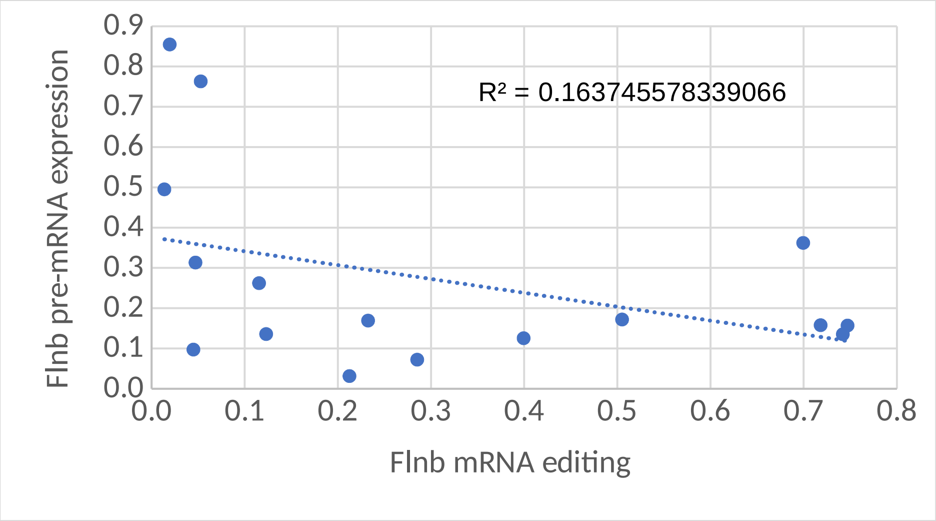

Supplemental Figure S3: Correlation between pre-mRNA expression and pre-mRNA editing or mRNA editing levels for the transcripts *Flna and Flnb,* respectively. Relative pre-mRNA expression levels for *Flna* (A, B) and *Flnb* (C, D) were correlated with the respective pre-mRNA and mRNA editing levels.

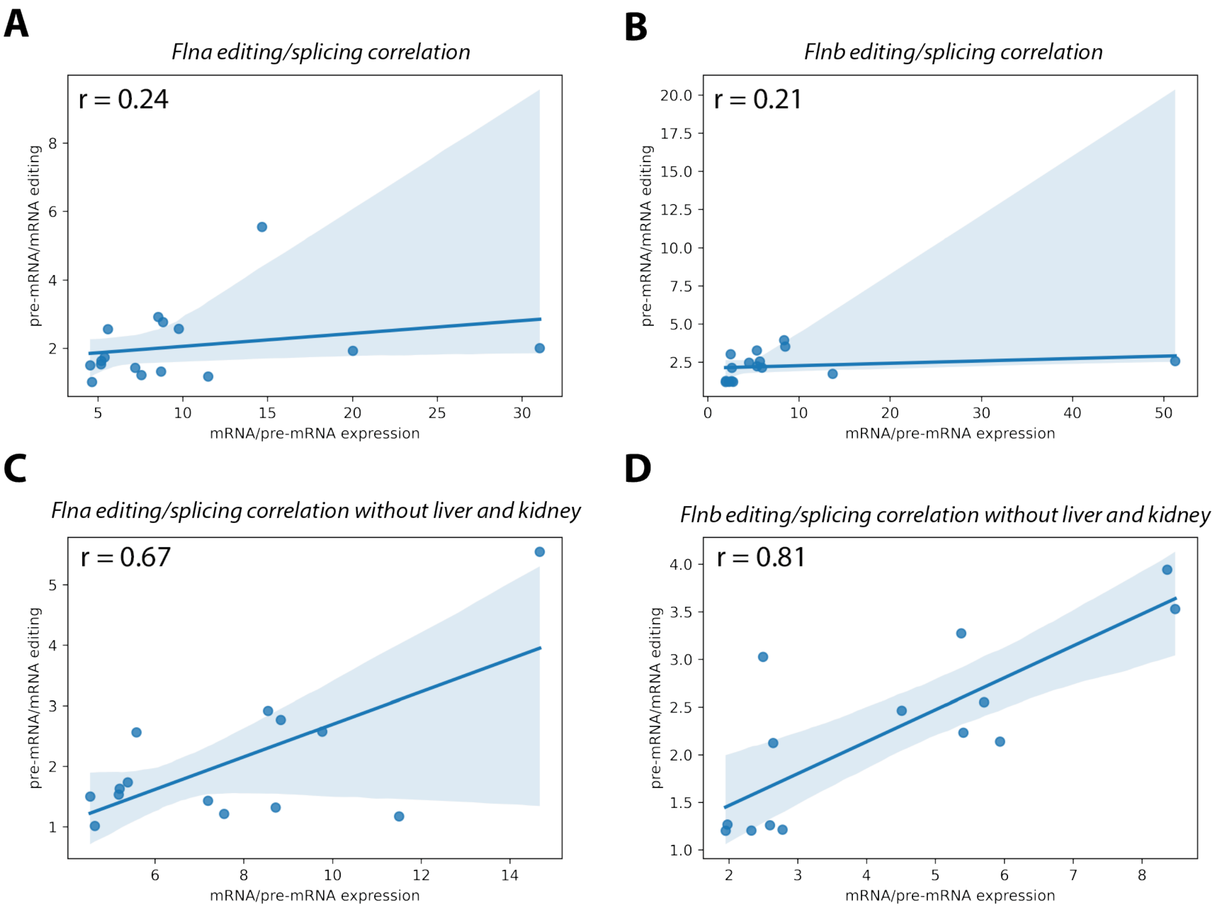

Supplemental Figure S4: *Flna* and *Flnb* editing levels correlate with pre-mRNA splicing across most mouse tissues. pre-mRNA and mRNA expression levels were determined using qPCR and splicing efficiencies were calculated (mRNA/pre-mRNA expression). Subsequently, splicing efficiencies and editing levels across mouse tissues were correlated for the same 16 tissues shown in main figure 3 (A, B). The same calculation was repeated for only 14 tissues without liver and kidney (C, D).

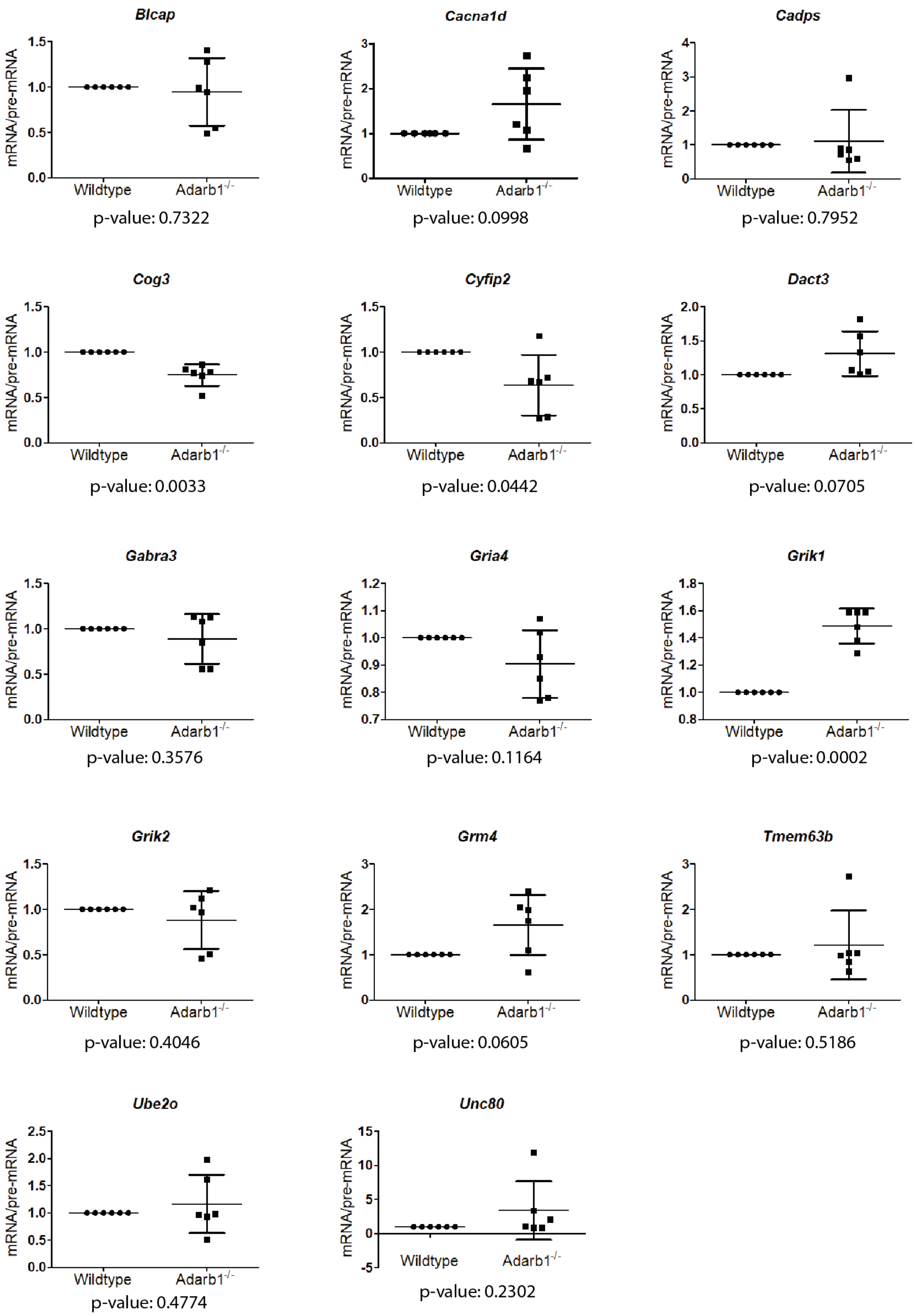

Supplemental Figure S5: Splicing efficiencies for several editing targets in wild-type and *Adarb1*$^{-/-}$ brain tissue. Pre-mRNA and mRNA expression levels were determined using qPCR and splicing efficiencies were calculated (mRNA/pre-mRNA expression).

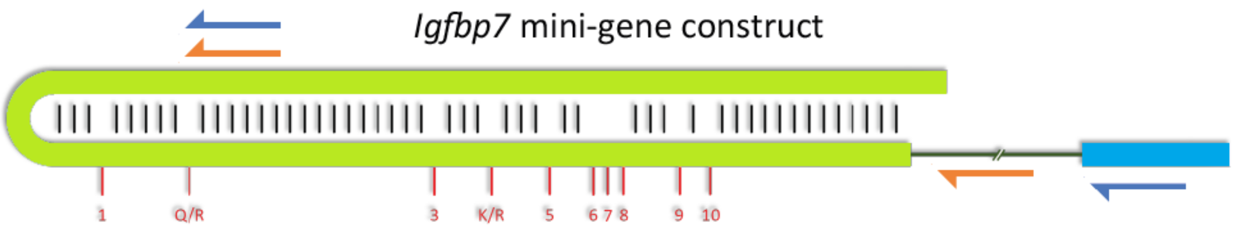

Supplemental Figure S6: Schematic of the *Igfbp7* mini-gene construct. In the construct *Igfbp7* exon 1 (green) including a part of the downstream intron is fused to the last 60 nucleotides of intron 1 plus exon 2 from the Adenovirus major late transcript (blue). Primers used to amplify the pre-mRNA and mRNA are drawn as orange and blue arrows, respectively. Editing sites are highlighted below the respective position in the exon.

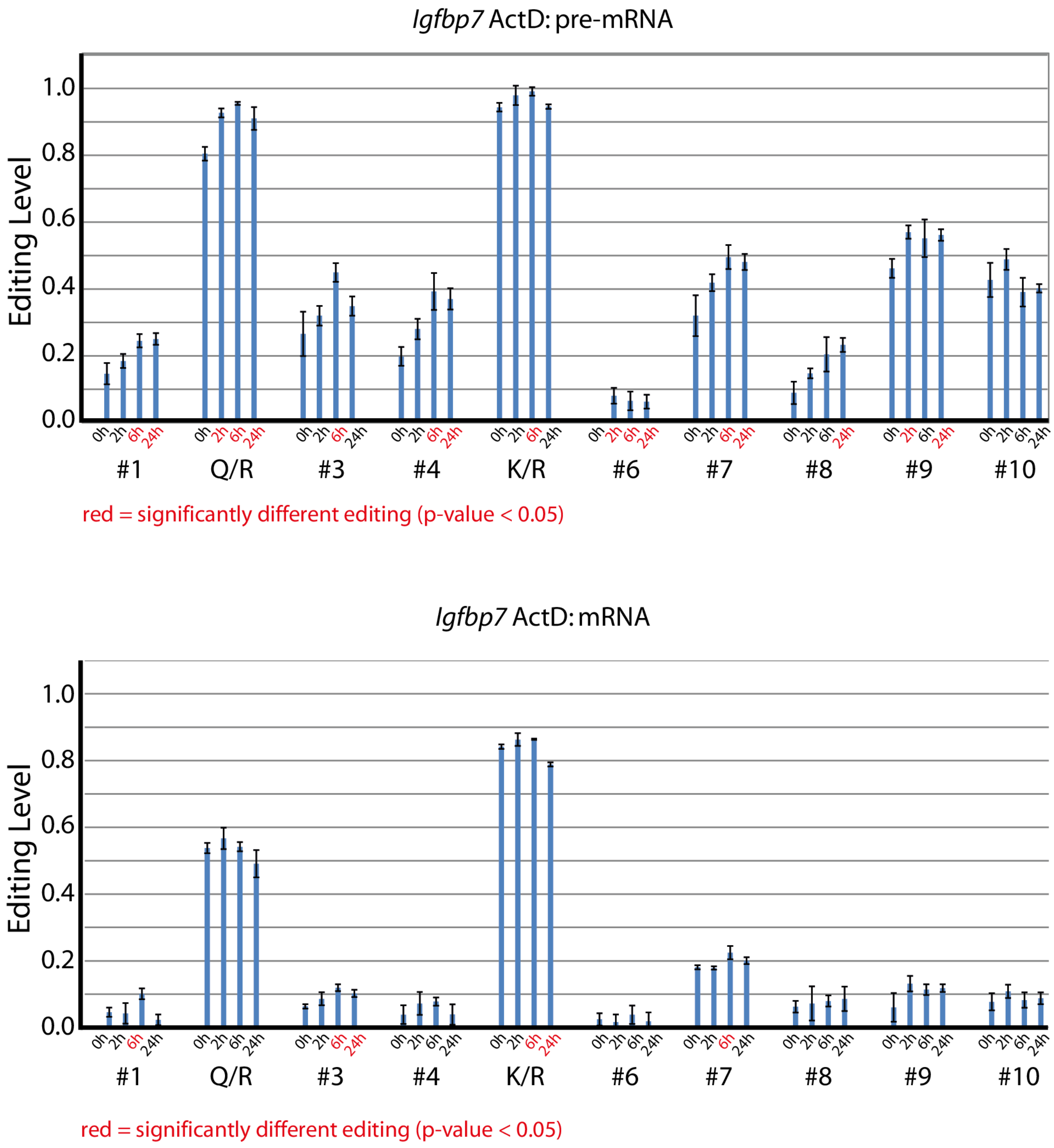

Supplemental Figure S7: *Igfbp7* editing levels increase upon stalling transcription in particular for the pre-mRNA (Detailed version of main figure 5 panel D). An *Igfbp7* construct was co-transfected with a plasmid expressing wild-type ADARB1 into HEK293 cells. Subsequently, transcription was blocked with Actinomycin D (ActD). Using Sanger sequencing, editing levels for pre- mRNA and mRNA were determined at time points 0h, 2h, 6h, and 24h after ActD treatment. n=3, error bars = standard deviation. Editing levels for time points marked in red are significantly different from the editing level in time point zero (p-value < 0.05).

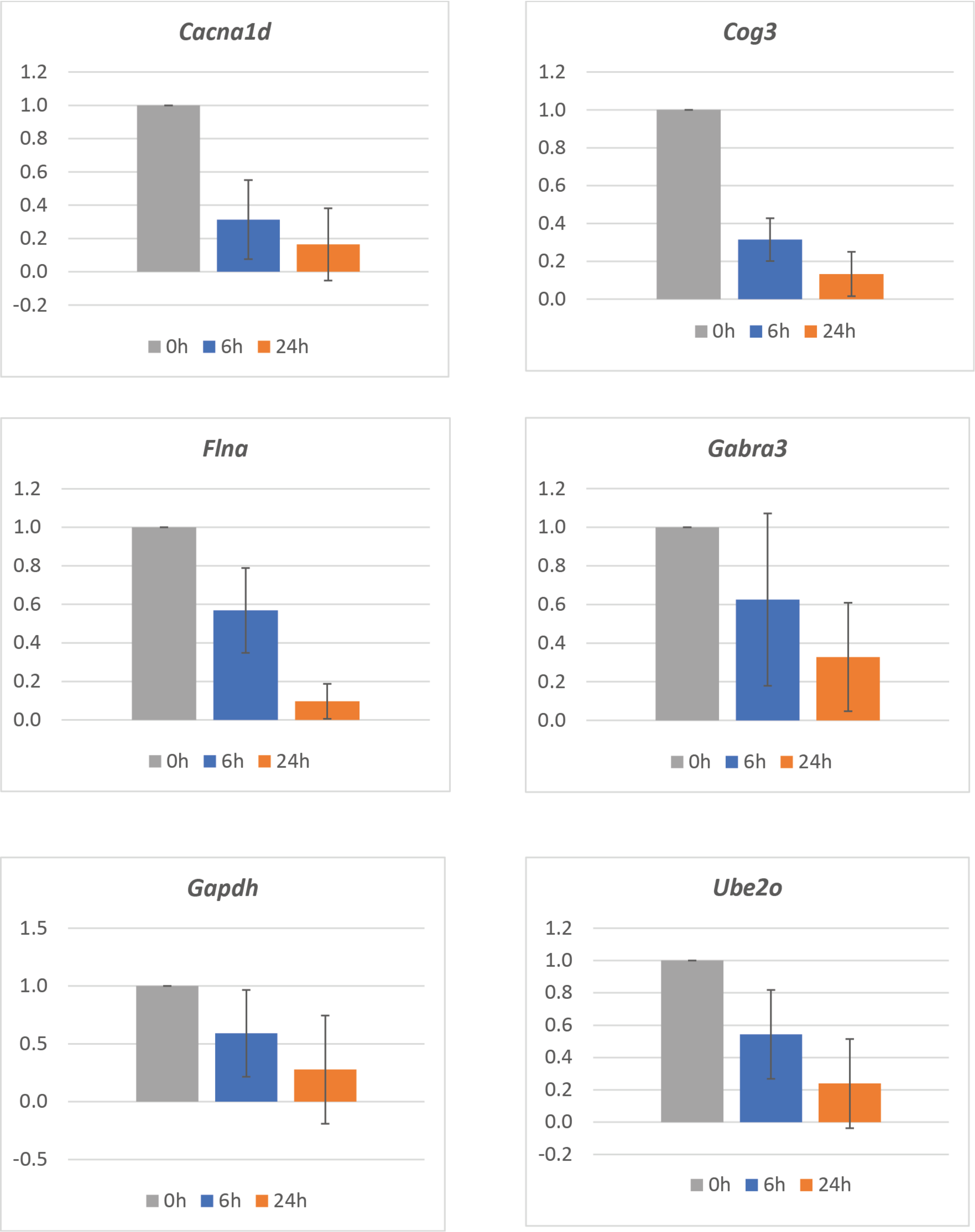

Supplemental Figure S8: Actinomycin D treatment in primary neuronal cells successfully blocks transcription as evidenced by reduced gene expression levels. Relative expression levels for six exemplary transcripts were determined using qPCR following Actinomycin D treatment at time points 0h, 6h, and 24h.

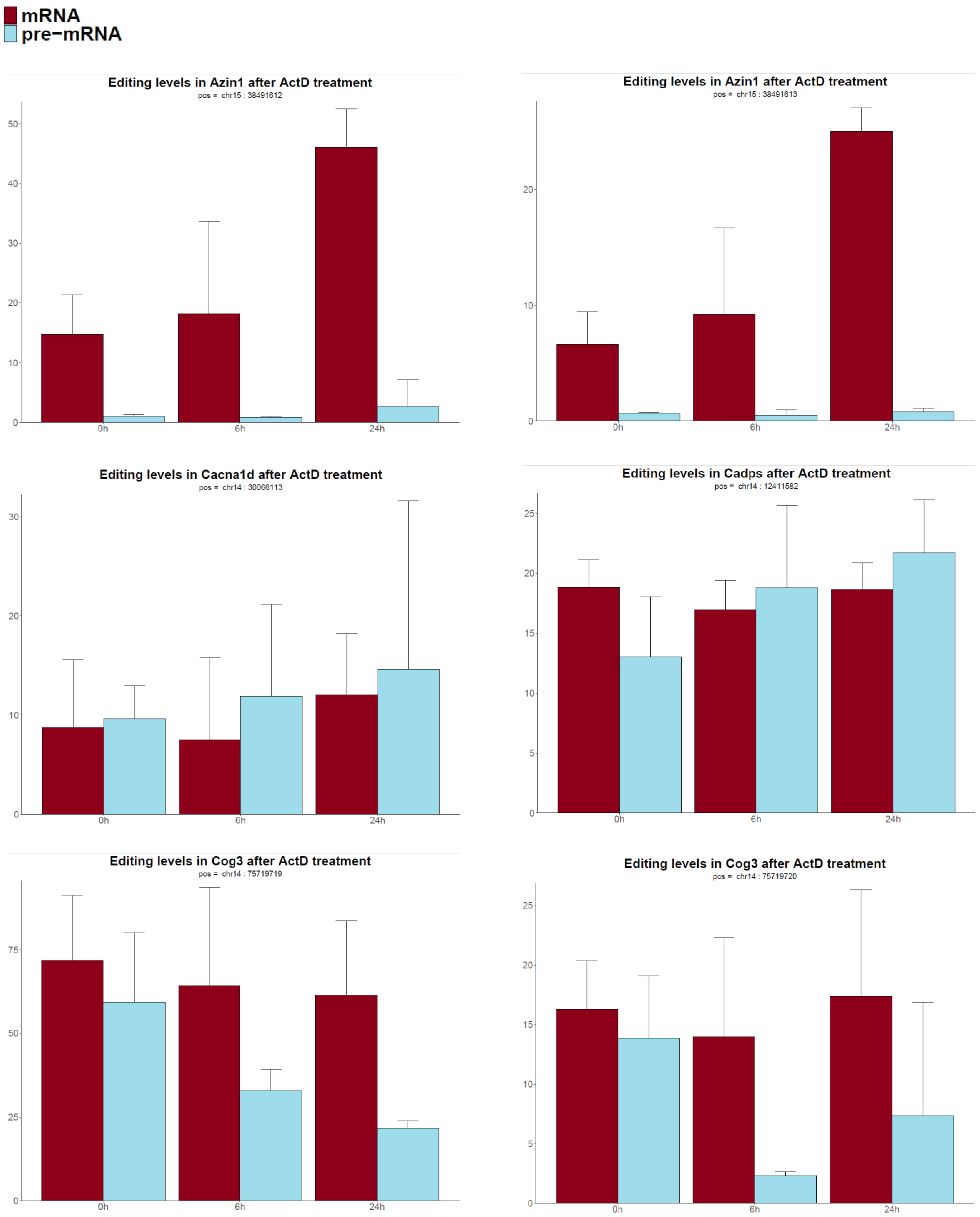

mRNA
pre−mRNA
Editing levels in Azin1 after ActD treatment
pos = chr15 : 38491612
0
10
20
30
40
50
0h
6h
24h
Editing levels in Azin1 after ActD treatment
pos = chr15 : 38491613
0
10
20
0h
6h
24h
Editing levels in Cacna1d after ActD treatment
pos = chr14 : 30066113
0
10
20
30
0h
6h
24h
Editing levels in Cadps after ActD treatment
pos = chr14 : 12411582
0
5
10
15
20
25
0h
6h
24h
Editing levels in Cog3 after ActD treatment
pos = chr14 : 75719719
0
25
50
75
0h
6h
24h
Editing levels in Cog3 after ActD treatment
pos = chr14 : 75719720
0
5
10
15
20
25
0h
6h
24h

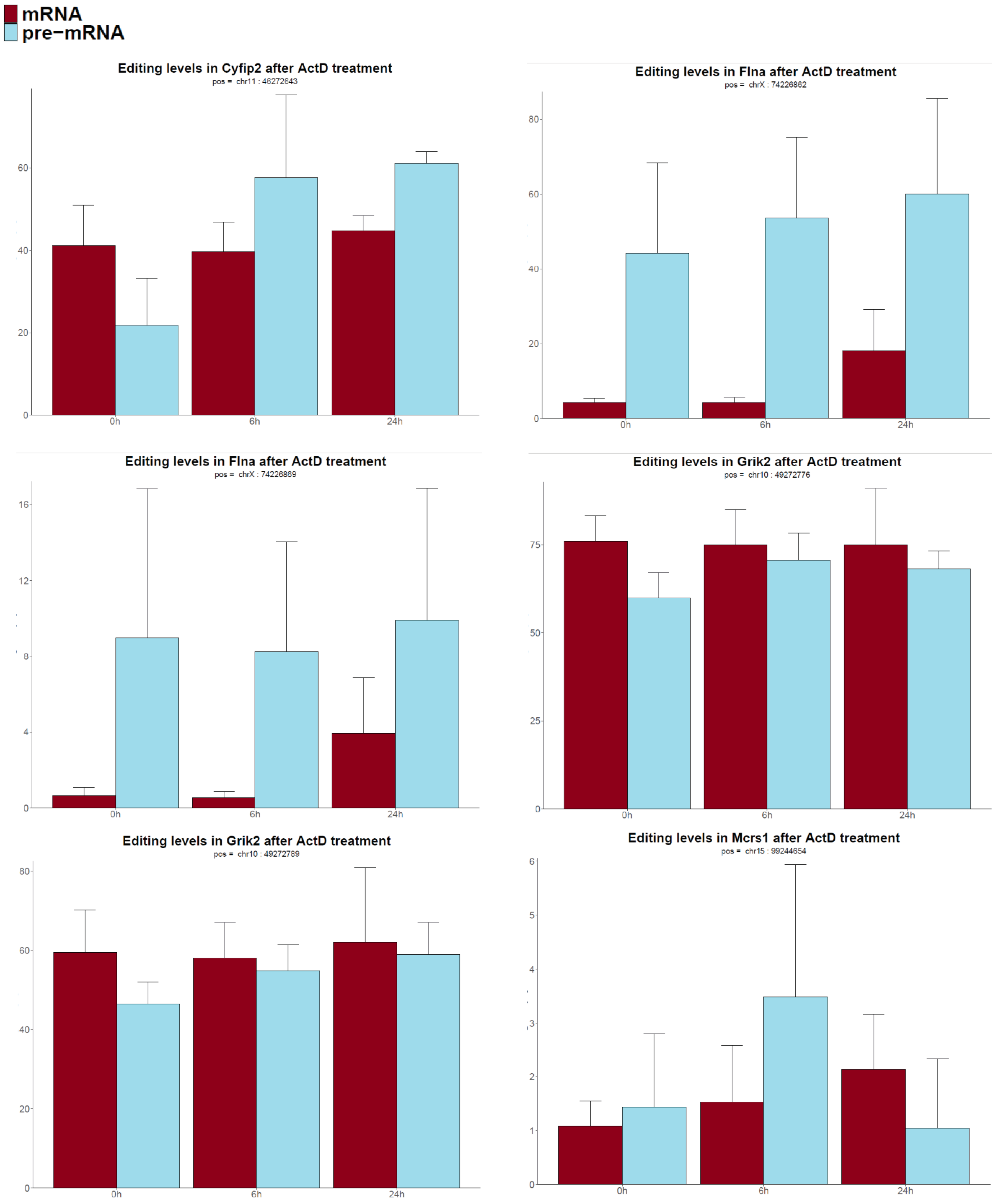

mRNA
pre−mRNA
Editing levels in Cyfip2 after ActD treatment
pos = chr11 : 46272643
0
20
40
60
0h
6h
24h
Editing levels in Flna after ActD treatment
pos = chrX : 74226862
0
20
40
60
80
0h
6h
24h
Editing levels in Flna after ActD treatment
pos = chrX : 74226869
0
4
8
12
16
0h
6h
24h
Editing levels in Grik2 after ActD treatment
pos = chr10 : 49272776
0
25
50
75
0h
6h
24h
Editing levels in Grik2 after ActD treatment
pos = chr10 : 49272789
0
20
40
60
80
0h
6h
24h
Editing levels in Mcrs1 after ActD treatment
pos = chr15 : 99244654
0
1
2
3
4
5
6
0h
6h
24h

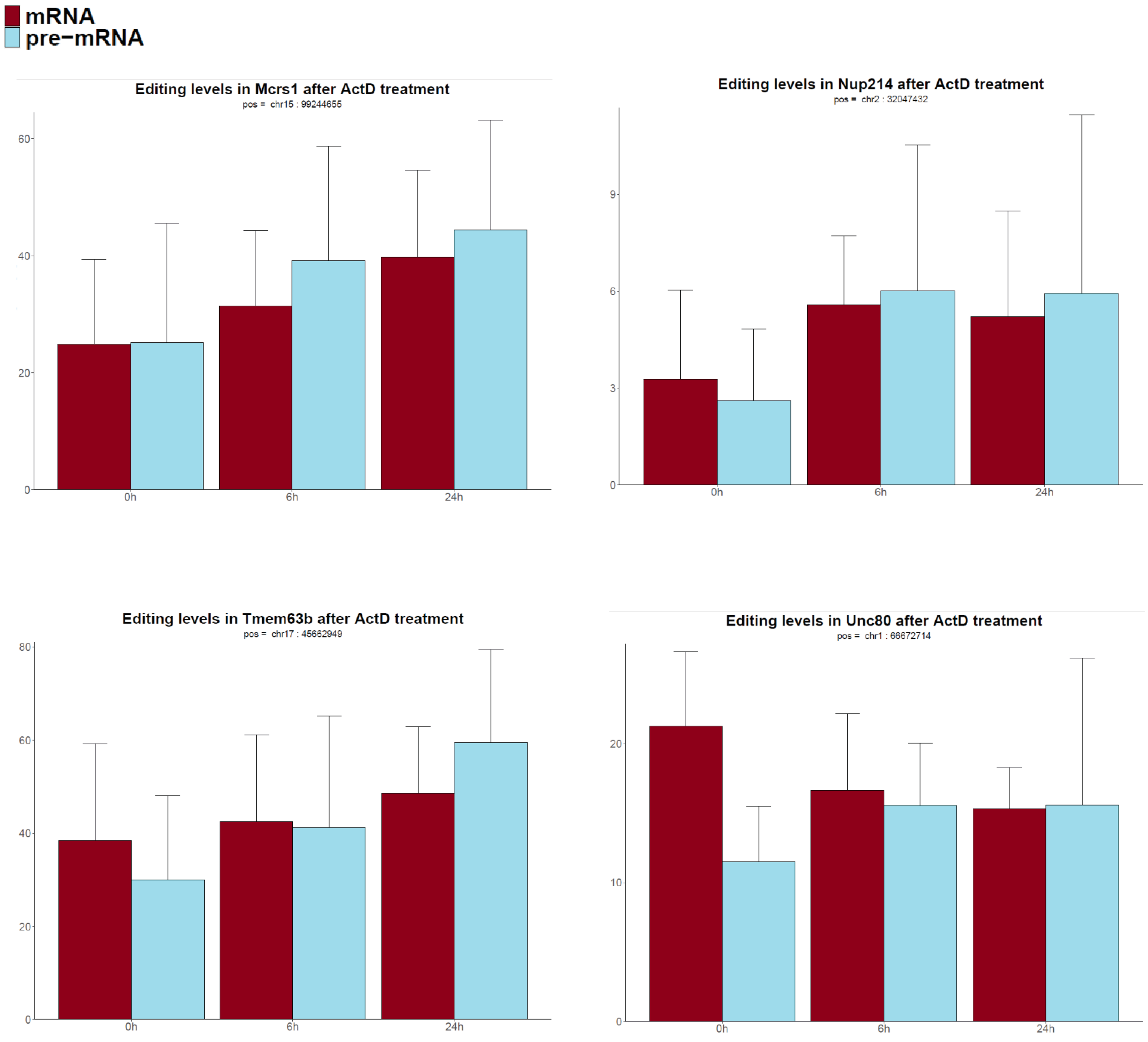

Supplemental Figure S9: Pre-mRNA and mRNA editing levels under conditions where transcription is blocked. RNA was isolated from primary neuronal cultures established from wild-type mouse embryos at e12.5 that were treated with Actinomycin D in order to stall transcription. Subsequently, pre-mRNA (light blue) and mRNA (dark red) editing levels were determined using amplicon-sequencing.

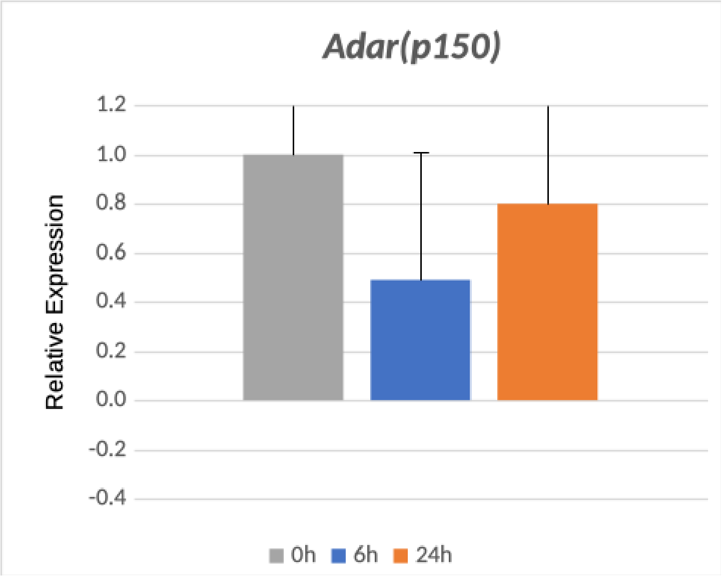

Supplemental Figure S10: *Adar*-p150 expression following Actinomycin D treatment. *Adar*-p150 expression in primary neuronal cultures treated with Actinomycin D was determined using qPCR.

Supplemental Materials and Methods

Expression of Adar-p150 following Actinomycin D treatment

RNA isolated from primary neuronal cells after Actinomycin D treatment was incubated with 20 units of DNaseI (New England BioLabs) at 37°C for 60 minutes. Subsequently, RNA was phenol-chloroform extracted. 100 ng of total RNA was reverse transcribed using random hexamer and oligo d(T) priming with LunaScript RT Master Mix Kit (New England BioLabs) following manufacturer's instructions. qPCR was performed using Luna Universal qPCR Master Mix (New England Biolabs). ΔcT values are relative to *beta-Actin*. Primer sequences are as follows: mouse *Adar-p150*: Fwd 5' – GGCACTATGTCTCAAGGGTTCA – 3', Rev 5' – AGCCGCCCTTTGAGAAACTCTA – 3', *Actin*: Fwd 5' – CTTTGCAGCTCCTTCGTTGC – 3'. Rev 5' – ACGATGGAGGGGAATACAGC – 3'.

Cloning of CMV/pGK minigene constructs

Either the Cytomegalovirus (CMV) or mouse phosphoglycerate kinase 1 (PGK) promoter was cloned into pGL3-Basic (Promega) using KpnI and XhoI. Subsequently, editing targets that had been cloned into pcDNA3.1 (Licht et al. 2016) were removed from pcDNA3.1 by restriction digest using XhoI and HindIII. Subsequently, the inserts were cloned into pGL3-Basic-CMV and pGL3-Basic-pGK, respectively. Successful cloning of previously sequence-validated inserts was confirmed by a miniprep followed by a restriction digest with XhoI and HindIII (New England BioLabs). Plasmid was purified with PureYield Plasmid Midiprep System (Promega).

Transfection, RNA isolation, RT-PCR, and Sanger Sequencing

24 h prior to transfection 3 × 10^5 tREx cells expressing hADAR2-Flag, 3 × 10^5 tREx cells expressing hADAR1p150-Flag, and 2 × 10^5 wt tREx cells were seeded on 6-well plates and then induced with 0.5 µg/mL doxycycline 12 h prior to transfection. The respective minigene plasmid was co-transfected with pHRL-TK using Lipofectamine-3000 (Thermo Scientific) following the manufacturer's instructions. To generate biological replicates, at least three independent transfections were made. For all constructs (except *Gabra3* constructs), 200 ng of minigene plasmid and 100 ng of pHRL-TK were transfected into either hADAR2-Flag expressing tREx cells (*Flna, Cyfip2, Gria2*) and wt tREx cells. For *Gabra3* constructs, 100 ng of minigene plasmid and 100 ng of pHRL-TK were transfected into both hADAR2-Flag expressing tREx cells and hADAR1p150-Flag expressing tREx cells, as well as wt tREx cells. 12 h after transfection, cells were washed with 1 mL PBS and given fresh medium. 36 h after transfection, cells were again washed with 3 mL PBS-T, then resuspended in 2 mL PBS. The cell suspension was split into 400 µL for a Western Blot to confirm ADAR expression, 100 µL for Dual-Luciferase Assay to confirm construct expression, and 1500 µL for RNA extraction. The Dual-Luciferase Assay was performed with Dual-Luciferase Reporter Assay System (Promega) according to manufacturer's guidelines. RNA was isolated with EchoLUTION Cell Culture RNA Kit (BioEcho) following manufacturer's instructions. To remove genomic DNA and plasmid DNA, total RNA was incubated with 2 µL BamHI-HF (New England BioLabs), 1 µL AluI (New England BioLabs), 1 µL MluCI (New England BioLabs), and 0.5 µL Murine RNase Inhibitor (New England Biolabs) at 37°C for 60

minutes, following by a digestion with 20 units of DNaseI (New England BioLabs) at 37°C for 60 minutes. Subsequently, RNA was phenol-chloroform extracted. Restriction endonuclease and DNaseI incubation was repeated as above, again followed by a phenol-chloroform extraction. 500 ng of total RNA were reverse transcribed using random hexamer priming and Maxima H Minus Reverse Transcriptase (Thermo Scientific) following manufacturer's instructions. A control without reverse transcriptase was included for every sample. To amplify products for Sanger sequencing, Taq-polymerase (New England BioLabs) was used with the following standard PCR protocol: 3 min at 94°C for 30 seconds, then 30 cycles of 94°C for 30 seconds, 58°C (for exon-exon primers) or 52.5°C (for exon-intron primers) for 30 seconds, 68°C for 30 seconds, followed by 68°C for 5 minutes. PCR reactions were subjected to gel electrophoresis on 1.5% agarose gels and PCR products were excised from the gel and purified with InnuPREP DOUBLEpure Kit (iST Immunoscreen Gmbh) following manufacturer's instructions. Sanger sequencing (Eurofins) was done using the eluted PCR products and a nested forward primer for all constructs. Editing levels at known sites were calculated using peak height given by SnapGene viewer, where the percentage of editing is calculated as the number of G divided by the sum of A plus G, or as the number of C divided by the sum of T plus C. For all constructs, universal primers were used with the following sequences: Universal Fwd 5'-GCCACTGTGCTGGATATCTG-3', Universal AdML exon 2 primer: Rev 5'-GAAAGACCGCGAAGAGTTTG–3', Universal AdML intron 2 primer: Rev 5'–GGGACAGGATAAGTATGAC–3'. Primer sequences used for Sanger sequencing are as follows: *Flna* constructs: Fwd 5'–GTCAAGTTCAACGAGGAGCAC–3', *Cyfip2* constructs: Fwd 5'–CAGCTGCAGGTGGTGC–3', *Gria2* constructs: Fwd 5'–CAAAGGAAGCCTTGCGACAC–3', *Gabra3* constructs: Fwd 5'–TGCTTACCATGACCACCTTG–3'

Supplemental References

Licht K, Kapoor U, Mayrhofer E, Jantsch MF. 2016. Adenosine to Inosine editing frequency controlled by splicing efficiency. *Nucleic Acids Res* **44**.
